# Sialylated human milk oligosaccharides program cognitive development through a non-genomic transmission mode

**DOI:** 10.1038/s41380-021-01054-9

**Published:** 2021-03-04

**Authors:** Jonas Hauser, Edoardo Pisa, Alejandro Arias Vásquez, Flavio Tomasi, Alice Traversa, Valentina Chiodi, Francois-Pierre Martin, Norbert Sprenger, Oksana Lukjancenko, Alix Zollinger, Sylviane Metairon, Nora Schneider, Pascal Steiner, Alberto Martire, Viviana Caputo, Simone Macrì

**Affiliations:** 1 Société des Produits Nestlé SA, Nestlé Research, Lausanne, Switzerland; 2 Centre for Behavioural Sciences and Mental Health, Istituto Superiore di Sanità, Rome, Italy; 3 Department of Physiology and Pharmacology “Vittorio Erspamer”, “Sapienza” University of Rome, Rome, Italy; 4 Donders Institute for Brain, Cognition and Behaviour, Departments of Psychiatry and Human Genetics, Radboud University Medical Centre, Nijmegen, The Netherlands; 5 Laboratory of Clinical Genomics, Fondazione IRCCS Casa Sollievo della Sofferenza, San Giovanni Rotondo, FG Italy; 6 National Centre for Drug Research and Evaluation, Istituto Superiore di Sanità, Rome, Italy; 7 Clinical Microbiomics, Copenhagen, Denmark; 8 Department of Experimental Medicine, “Sapienza” University of Rome, Rome, Italy

**Keywords:** Psychology, Neuroscience, Genetics

## Abstract

Breastmilk contains bioactive molecules essential for brain and cognitive development. While sialylated human milk oligosaccharides (HMOs) have been implicated in phenotypic programming, their selective role and underlying mechanisms remained elusive. Here, we investigated the long-term consequences of a selective lactational deprivation of a specific sialylated HMO in mice. We capitalized on a knock-out (KO) mouse model (B6.129-*St6gal1*^*tm2Jxm*^/J) lacking the gene responsible for the synthesis of sialyl(alpha2,6)lactose (6′SL), one of the two sources of sialic acid (Neu5Ac) to the lactating offspring. Neu5Ac is involved in the formation of brain structures sustaining cognition. To deprive lactating offspring of 6′SL, we cross-fostered newborn wild-type (WT) pups to KO dams, which provide 6′SL-deficient milk. To test whether lactational 6′SL deprivation affects cognitive capabilities in adulthood, we assessed attention, perseveration, and memory. To detail the associated endophenotypes, we investigated hippocampal electrophysiology, plasma metabolomics, and gut microbiota composition. To investigate the underlying molecular mechanisms, we assessed gene expression (at eye-opening and in adulthood) in two brain regions mediating executive functions and memory (hippocampus and prefrontal cortex, PFC). Compared to control mice, WT offspring deprived of 6′SL during lactation exhibited consistent alterations in all cognitive functions addressed, hippocampal electrophysiology, and in pathways regulating the serotonergic system (identified through gut microbiota and plasma metabolomics). These were associated with a site- (PFC) and time-specific (eye-opening) reduced expression of genes involved in central nervous system development. Our data suggest that 6′SL in maternal milk adjusts cognitive development through a short-term upregulation of genes modulating neuronal patterning in the PFC.

## Introduction

Human milk is without a doubt the best source of nutrition for the infant. Beside well-known long-term benefits [1, 2], human milk is essential to support optimal neurodevelopment and other physiological processes [3]. In particular, breastfeeding promotes brain [4] and cognitive [5, 6] development on a structural and functional level [7–9]. These effects have been proposed to relate to specific bioactive breastmilk nutrients which program the development of cognitive capabilities [4–6] and their neurobiological determinants [3].

For example, children who were not breastfed or breastfed for short periods have a lower verbal intelligence quotient at 30 years of age compared to age-matched controls fed exclusively breastmilk for 6 months or more [10]. These data support the hypothesis that specific factors linked to breastfeeding, ranging from mother–infant bond to specific bioactive nutrients, are key for proper brain and cognitive development. Concerning the latter, human milk oligosaccharides (HMOs) represent the third most abundant class of nutrients of human milk (beside lactose and lipids) [11]. Differently from other nutrients, HMOs do not represent major energy sources. Sialylated HMOs (i.e., oligosaccharides containing sialic acid—Neu5Ac—residues in their structure), in particular, may constitute a primary regulator of cognitive development as they represent the principal source of Neu5Ac to the lactating offspring [12]. While the precise mechanisms underlying its programming role at the level of the central nervous system (CNS) are still elusive, Neu5Ac has been proposed to exert its effects through acting as building block or binding element in molecules that play a role in neurodevelopment: gangliosides and polysialyllated neural cell adhesion molecules (polySia-NCAM). While gangliosides are fundamental in myelination [13], neuronal circuits [14], neurogenesis, synaptogenesis, and memory formation [12], polySia-NCAMs are involved in numerous physiological (e.g., neuroinflammation, neurotoxicity, neuronal growth [13]) and neurodevelopmental processes, including the maturation and differentiation of neural cells as well as long-term potentiation (LTP) [13, 15, 16].

During infancy, due to a low activity of the gene responsible for its synthesis, the endogenous availability of Neu5Ac is not sufficient to guarantee the proper maturation of the CNS [12]. Nutritional needs of Neu5Ac during lactation are therefore met by the sialylated HMOs present in maternal milk, the most abundant being sialyl(alpha2,6)lactose (6′-sialyllactose (6′SL)) and sialyl(alpha2,3)lactose (3′-sialyllactose (3′SL)), accounting for ~75% of breastmilk sialic acid content [17]. Interestingly, milk replacements (e.g., bovine and formula) have no or much lower concentrations of sialylated HMOs, and may thus fail to cover the needs of Neu5Ac [9]. This may explain the lower concentrations of Neu5Ac, brain gangliosides, and polySia-glycoproteins observed in the frontal cortex and gray matter of formula-fed vs. breastfed infants [7].

Beside the correlational data observed in humans [4–6], the functional role of Neu5Ac has been investigated in numerous preclinical studies. Wang et al. demonstrated that the supplementation of Neu5Ac in piglets improves learning and memory capabilities during treatment [18]. Oliveros et al. [19] performed a cross-fostering study in rodents, in which 3-days old rats were transferred to a foster dam that had delivered its offspring 16 days before, resulting in pups receiving a milk deficient in numerous nutrients including sialylated HMOs. These rats were then supplemented with dietary Neu5Ac or 6′SL and, compared to rats nurtured by the foster dam, exhibited improved in vivo LTP and novel object recognition memory [19]. Improved cognition in response to sialyllactose administration has also been observed in preterm pigs [20].

Notwithstanding the aforementioned studies, evidence in support of a neurodevelopmental programming role of sialylated HMOs is only indirect since no available study has tested whether the time-specific (during lactation) and selective deprivation of a key HMO (6′SL) relates to cognitive impairments (memory, attention, sensorimotor gating, and perseverative behavior) in adulthood. Here, we addressed this question through a balanced cross-fostering design in which neonate control mice (wild-type (WT)) were fostered to knock-out dams (B6.129-*St6gal1*^*tm2Jxm*^/J, hereafter KO)—genetically engineered to provide 6′SL-deficient milk [8]—that delivered their offspring on the same day. Once adult (postnatal day >70), these mice and their relative control subjects (WT offspring in-fostered to WT dams, and KO offspring in- and cross-fostered to WT and KO dams), were assessed for their cognitive performance and, to identify potential causative mechanisms, for the expression of genes responsible for the development of the biological substrates serving these functions. Additionally, to gain further insights into the biological determinants of the observed phenotype, we investigated their plasma metabolomics and fecal microbiota composition. The latter has recently emerged as an important regulator of CNS development and activity via a direct link between the gut and the brain (gut-brain axis [21, 22]). The microbiota, a key component of the gut-brain axis, is capable of modulating CNS activity through the autonomic nervous system, the enteric nervous system, the immune system, and the bacterial metabolites. Specifically, the gut microbiota acts through the regulation of metabolites, neurotransmitters (e.g., serotonin and catecholamines) and their building blocks (e.g., tryptophan) [23, 24]. Based on these considerations, we selectively quantified those microbiota species involved in tryptophan metabolism (e.g., in its conversion into catabolites like indolelactate or indole-3-carboxylate) and CNS development.

## Materials and methods

### Animals and rearing conditions

Adult WT B6.129 and heterozygous (HZ) B6.129-*St6gal1*^*tm2Jxm*^/J mice (*Mus musculus*, JAX stock #006901) breeding pairs (four males and four females and three males and four females, respectively) were purchased from a commercial breeder (The Jackson Laboratory, Bar Harbor, Maine, USA [25]). Upon arrival, same-sex mice were housed in same-sex groups of two to three in type-1 polycarbonate cages (33.0 × 13.0 × 14.0 cm) equipped with sawdust bedding, shredding nest material (Nestlet squares, 2 Biological Instruments S.N.C., Besozzo, Italy), metal top and ad libitum water and food pellets (Mucedola, Settimo Milanese, Italy). Mice were maintained on a reversed 12-h-light–dark cycle (lights on at 18:00) in an air-conditioned room (temperature 21 ± 1 °C and relative humidity 60 ± 10%). Two weeks after arrival, breeding triads (one male, two females) were formed. After 2 weeks of mating, male mice were removed, and females were housed individually in standard type-1 cages. Females were checked daily for delivery and the day in which they gave birth was designated as postnatal day 0 (PND0). Apart from cage cleaning once a week, dams and their offspring were kept undisturbed until weaning (on PND28). At weaning, male and female mice were separated and housed in same-sex same-litter cages and marked through ear clipping. Tail biopsies were used to ascertain the presence of WT and KO male and female mice (see Supplementary Information, for details on the genotyping procedures) to be used in the experiments.

**Fig. 1:**
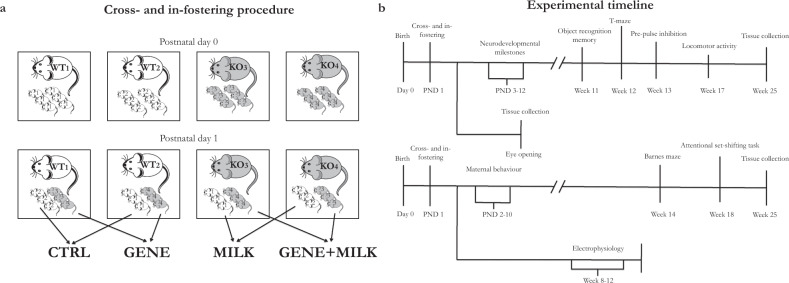
Neonatal manipulations and experimental timeline. **a** Cross- and in-fostering procedures: 1 day after birth, dams remained in their home cages while offspring were transferred from their original cages to those housing their foster dams. At the end of fostering procedures, litters consisted of wild-type (WT) and knock-out (KO) mice in a 1:1 ratio. **b** Experimental time-schedule and allocation of the four study cohorts to the different assessments. After the cross- and in-fostering procedures, experimental subjects were allocated to two study cohorts. Subjects in cohort 1 (upper line, *N* = 10 per group) were tested for neurodevelopmental milestones (Supplementary Fig. 3), object recognition memory, T-maze, prepulse inhibition, general locomotion and were then used for tissue collection, specifically brain and gut samples (prefrontal cortex, hippocampus, striatum and cecum content, respectively) at 25 weeks of age. An independent cohort was used to collect samples at eye-opening for brain RNA-seq, plasma metabolic phenotyping and fecal microbiome analyses (*N* = 12 per group). Subjects in cohort 2 (lower line, *N* = 10 per group) were exposed to the Barnes maze, to the attentional set-shifting task, and then used for tissue collection specifically brain and gut samples (prefrontal cortex, hippocampus, striatum, and cecum content, respectively) at 25 weeks of age. An independent cohort (*N* = 3–5 per group) was used to evaluate long-term potentiation (LTP) in hippocampal slices (electrophysiology experiment). To avoid litter effects, each group in each cohort consisted of mice born to different dams; to limit test battery effects, the presentation of test paradigms was scheduled based on invasiveness-level considerations, wherein the most invasive test was performed at the end of the battery. Active maternal care was assessed, during the first 10 days of life, in dams of the cohort 2.

Twenty-eight WT and 28 KO female mice have been mated with 14 WT and 14 KO male mice, respectively. Out of this batch, 24 WT and 24 KO dams gave birth to a viable litter. Day of birth was designated as PND0. The fostering procedure (see Fig. 1a for details), performed on PND1 between 10:00 and 13:00, required the use of four dams (two WT and two KO) at the same time. We first removed the dams from their cage and then sexed and marked the offspring through toe tattoo ink puncture [26]. Pups were then moved to the cage housing the foster dam and covered with sawdust. Each dam nurtured a mixed litter composed of non-biological WT and KO male and female offspring (1:1 ratio among all variables whenever possible).

At weaning (PND28), male mice reared to the same dam were transferred together (two or three mice per cage) to standard type-1 polycarbonate cages (33.0 × 13.0 × 14.0 cm) and kept in the same conditions described above. These procedures resulted in four experimental groups of male mice: CTRL (in-fostered WT offspring, *N* = 37), consisting of WT mice receiving milk with 6′SL; MILK (cross-fostered WT offspring, *N* = 35), consisting of WT mice receiving milk without 6′SL; GENE (cross-fostered KO offspring, *N* = 37), consisting of KO mice receiving milk with 6′SL; GENE + MILK (in-fostered KO offspring, *N* = 37), consisting of KO mice receiving milk without 6′SL. Developing offspring were subdivided into two equally sized cohorts (*N* = 10 per group per cohort) tested for different behavioral parameters and sampled for postmortem analyses in adulthood; an additional cohort (*N* = 12 per group) was used to collect plasma, fecal, and brain samples at eye-opening for subsequent brain tissue RNA-seq, plasma metabolic phenotyping, and fecal microbiome analyses (Fig. 1b). A fourth batch of male mice exposed to identical cross-fostering procedures (*N* = 3–5 mice per group) was used to assess electrophysiological correlates of memory performance (LTP in hippocampal slices). To avoid litter effects, we never allocated littermates reared from the same dam to the same test battery. Specifically, all subjects contributing to a given experimental paradigm derived from different dams. Following this allocation, experimental subjects were randomly assigned to behavioral testing and postmortem analyses. Blinding to treatments in postmortem analyses was ascertained by providing the experimenters with samples that were labeled with codes masking the identity of the subjects. With respect to behavioral outcomes: ORMT, PPI, Barnes maze, and general locomotion were quantified by automated software which, by definition, is blind to treatments; for T-maze, the experimenter conducting the test received the mouse from another experimenter who guaranteed blinding; for maternal care observations and ASST blinding was not possible.

### Behavioral tests

#### Maternal behavior

After the cross-fostering procedure, we analyzed maternal behavior of eight WT and ten KO dams between PND 2 and 10. Maternal care was daily observed for three 1-h sessions. The first two sessions were conducted during the dark phase, while the last daily session was conducted during the light phase, starting, respectively, at 9:00, 14:00, and 18:00. Each session lasted 1 h in which we scored 20 instantaneous samples (interspaced by 3-min intervals) for each dam. In accordance with our previous studies [27], we scored the following behaviors: active maternal care, passive maternal care, and self-maintenance.

#### Neurodevelopmental milestones (PND3 and PND7)

Mice were evaluated for the onset of neurodevelopmental milestones through the administration of Fox scales [28]. Specifically, we evaluated the following reflexes: righting (mouse capability to turn over with all four paws when placed on its back); grasping (the mouse is placed on a wire-mesh grid which is progressively tilted until the subject harmlessly falls on a soft surface, positioned 2 cm below the grid); and cliff aversion (measuring the capability to withdraw from a perceived visual cliff, data not shown). Pups’ bodyweight and body length were constantly monitored throughout development.

#### Object recognition memory test (ORMT, week 11)

Mice were tested individually in an opaque dark familiar arena (40 × 40 × 40 cm) under indirect dim light. As described elsewhere [29], the test consists of a habituation phase (10 min) and two 10-min test trials, conducted 60 min and 24 h after habituation. Twenty-four hours before habituation, subjects were familiarized with the arena during a 30-min pre-exposure session. This session was video-recorded through a camera (Sony Handycam DCR-SX21E, Tokyo, Japan). Absolute levels of locomotion (distance moved throughout the 30-min test session), and percent time spent in the periphery of the open field (the mouse body center within 5.5 cm from the walls) were quantified offline through an automated tracking software (ANY-maze^®^, Stoelting Co., Wood Dale, IL, USA). During habituation, mice were exposed to two identical unfamiliar objects (A). During test trials, one of the two familiar objects (A) was replaced by unfamiliar objects: an object (B) 60 min after habituation and an object (C) 24 h after habituation. An exploration ratio, calculated as the time exploring the novel object divided by the time exploring both objects, was used to measure novel object exploration. An exploration ratio higher than 50% indicates a preference for the novel object. A camera (Sony Handycam DCR-SX21E, Tokyo, Japan) mounted above the arena recorded each trial and videos were analyzed offline by a human operator (intra-rater reliability coefficient 0.97, *p* < 0.05) through dedicated software (“The Observer XT”, Noldus, Wageningen, The Netherlands).

#### T-maze (week 12)

Animals were screened for perseverative behaviors in the T-maze test (see [30, 31] for details), an apparatus consisting of three equally sized arms (14.5 × 8 cm). Ten sessions, consisting of two choice trials, were performed during five consecutive days (two sessions per day). Each session started with the mouse positioned in the start compartment, facing the wall of the apparatus; then, the subject was allowed to explore the apparatus for 2 min. Upon entering one of the two alternative arms, such instance was scored as the first choice, the door was closed, and the subject was gently relocated in the start compartment to perform a second choice. If the subject entered the arm opposite to the previously chosen one, an instance of alternation was scored. The percentage of alternations was computed as the number of alternations divided by the number of completed sessions × 100. Data were collected and scored manually. A trained observer, blind to treatment, recorded both individual choice and latency to reach the chosen arm.

#### Prepulse inhibition (PPI, week 13)

PPI was conducted, as described elsewhere [30, 32], in an apparatus (Med Associates Inc. St Albans, VT, United States of America) consisting of an acoustic stimulator (ANL-925) and a platform with a transducer amplifier (PHM-250-60), positioned in a foam-lined isolation chamber (ENV-018S). Experimental data were acquired through dedicated software (SOF-815). During habituation, each mouse was positioned individually inside the startle chamber, and left undisturbed for 5 min. On the following day, mice were exposed to white noise (62 dB) for 5 min; after acclimation, mice were then exposed to a sequence of ten “startle” trials (pulses of 120 dB) interspaced by an average inter-trial interval of 15 s (block I). Then, a 16-min long session started (block II). This session entailed 56 trials comprising four different types of trial that were presented in a pseudorandomized order. Trials were defined as follows: prepulse alone, prepulse plus pulse, startle alone, and no stimulation. The intensity of the prepulse was 74, 78, 82, or 84 dB. Following the prepulse, a startle stimulus (40-ms long white noise, 120 dB of intensity) was presented. The galvanic response was considered as the dependent variable and was measured 65 ms after the onset of the startle. PPI was measured as PPI = [(A − B)/A × 100], wherein A is the Galvanic reflex registered after the startle stimulus alone, and B is the reflex registered in response to the startle in prepulse plus pulse trials.

#### Barnes maze (weeks 14–16)

The test was performed as described in [33]. Briefly, mice exposed to a bright light (85 lux) on a circular white arena (92 cm diameter, elevated 93 cm above the floor) were required to locate a rectangular escape box (7 × 37 × 9 cm) located underneath one of 20 holes (target hole, diameter 5 cm). To avoid position bias, the position of the escape box was randomized between subjects. The experimental protocol consisted of one day of habituation (two 1-min exploration trials), five days of training and two probe trials, conducted 24 hours or seven days after the last training session. During training (days 2–5), mice were exposed to two consecutive trials interspaced by a 10-min interval. Trial ended when the animal located the escape box, or after 3 min, when an operator gently directed the mouse to the right hole. Once located, the mouse was kept in the escape hole for one minute. Probe trials consisted of a 90 s free exploration during which the escape box was removed, and all the holes, including the target, were blocked. Memory, during the probe test, was evaluated through the automated scoring (“The Ethovision”, Noldus, Wageningen, The Netherlands) of the time spent in the target zone. During the probe test, we also evaluated general locomotion in terms of distance moved throughout the 90 s test session.

#### General locomotion (week 17)

Locomotor activity was evaluated over five consecutive days in the home cages through an automated scoring device (ACTIVISCOPE system, NewBehaviour Inc., Zurich, Switzerland) described in [34].

#### Attentional set-shifting task (weeks 18–23)

We adopted the attentional set-shifting task, originally developed by Birrell and Brown [35] and modified by Colacicco et al. [36]. The procedure (detailed in [37]) was conducted in an opaque PVC U-shaped box (45 × 30 × 15 cm) equipped with two identical choice compartments (15 × 15 cm). A cylindrical food cup (40 mm diameter, 35 mm high) in each choice compartment was baited with a small piece of cereal (30 mg; Honey Nut Loop, Kellogg, Battle Creek, MI, USA), covered with a layer of scented digging medium (20 mm, see Supplementary Table 1). The presence or absence of food reward in a cup was indicated by either tactile (type of digging medium) or olfactory stimuli (scent of the digging medium). Mice were requested to acquire a rule associated with the reward and then to disregard such rule in favor of a new one upon attainment of the learning criterion (eight correct responses out of ten consecutive trials, see Supplementary Information and Supplementary Table 1 for details). Using this test, we obtained information about the following stages: simple discrimination; compound discrimination (CD); CD reversal; intra-dimensional shift; and extra-dimensional shift. We measured the number of trials and errors until criterion. Each mouse was tested over a period of five days, during which it completed all the stages of the task. During each week, we tested a maximum of six subjects. Therefore, six weeks were required to complete the execution of the ASST test in all subjects, which were tested in a randomized and fully counterbalanced order.

### Brain and blood sampling

Brain samples have been collected at eye-opening (PND 13–15) and at 24–25 weeks of age. Mice were rapidly decapitated, blood was collected, and brain was quickly removed and dissected on ice. At both ages, we collected infralimbic and prelimbic cortex (hereafter prelimbic medial prefrontal cortex (PFC)), and right and left CA1 and CA3 regions of the hippocampus (HPC). To precisely dissect the aforementioned regions, we used the mouse brain atlas as reference [38]. Brain samples were put in cryotubes (Thermo Scientific Nunc Cryotubes Vials), flash frozen and stored at −80 °C. Trunk blood samples were collected in tubes (Sarstedt Microvette^®^) containing anticoagulants (EDTA), then centrifugated at 4 °C for 15 min at 4000 rpm for plasma extraction. Plasma samples were stored at −80 °C. Brain samples were used for RNA-seq analyses. Blood samples were used for metabolic phenotyping analyses.

### Gut sampling

Gut samples were collected from all the experimental groups at eye-opening (PND 13–15) and at 24–25 weeks of age. Mice were rapidly decapitated. The intestine was separated from the mesentery, clamping the gut after the stomach and cutting its end before the anus. Then, we collected cecum content. Samples were put in cryotubes (Thermo Scientific Nunc Cryotubes Vials) flash frozen and stored at −80 °C and later used for microbiota analyses.

### Electrophysiology experiments

LTP experiments were performed, as detailed in [39] on hippocampal slices collected between 8 and 12 weeks of age. Extracellular fEPSPs were recorded in stratum radiatum of the CA1 area after stimulation of Schaffer collaterals. Signals were acquired with a DAM-80 AC differential amplifier (WPI) and analyzed with the LTP program [40]. LTP was induced by a theta-burst stimulation (TBS) consisting in two trains of five sets of bursts (four stimuli, 100 Hz) with an interburst interval of 200 ms and a 20 s interval between each train. Slope values were normalized, taking the average of the baseline values to be 100%. Synaptic transmission was recorded for 60 min after TBS and 10 min of stable baseline recordings preceded LTP induction. Changes in fEPSP slope in the last 10 min of recording were expressed as percentage changes with respect to the average slope of the fEPSP measured during the 10 min that preceded the TBS. Curve fittings were obtained by using GraphPad Prism software (version 6.05, GraphPad Software, San Diego, California, USA).

### Gene expression analyses

#### Total RNA extraction

Total RNA was extracted using the Agencourt RNAdvance Tissue Kit (Beckman Coulter): Lysis was done in 450 µl. Quantification was performed using Quant It Ribogreen assay (Life Technologies) and quality control (QC) assessment was performed using Standard sensitivity RNA kit on Fragment Analyzer 96 (Agilent). Details on Sample Libraries preparation and sequencing are detailed in the Supplementary Information and Supplementary Fig. 1).

#### Gene expression data analysis

Raw counts were obtained from sequences by mapping to the mouse reference genome using STAR v.2.5.3 [41] and counting using htseq-count v.0.6.1 [42]. Features were filtered to remove lowly expressed genes by selecting only genes with at least five reads in at least eight samples. Filtering was performed on the CPM values which take the library size into account: a threshold of 2.427 on the CPM values. We also discarded genes with no annotation. We kept 11,523 features with these filtering criteria. Trimmed Mean of M-value normalization [43] was used to account for composition bias between libraries. Differential expression analysis between different groups was performed using edgeR v3.20.9 [44]. Empirical Bayes methods are used to moderate the degree of over dispersion. We further extended the negative binomial model with quasi-likelihood method to account for gene-specific variability. Differential expression was tested by quasi-likelihood *F*-tests and resulting *p* values were adjusted for multiple testing using Benjamini and Hochberg method (BH). Pathway analysis tests for over-representation of the differentially expressed genes in the gene ontology GO and Kyoto Encyclopedia of Genes and Genomes (KEGG) pathways. We extracted differentially expressed genes (FDR < 5%) and conduct overlap tests. Obtained *p* values were adjusted for multiple testing using BH.

### Metabolomics

#### Sample preparation

Samples were prepared using the automated MicroLab STAR^®^ system from Hamilton Company. Samples were extracted with methanol under vigorous shaking for 2 min (Glen Mills GenoGrinder 2000) followed by centrifugation. The resulting extract was divided into five fractions: two for analysis by two separate reverse phase Ultrahigh Performance Liquid Chromatography-Tandem Mass Spectroscopy (RP/UPLC-MS/MS, see Supplementary Information for details) methods using positive ion mode electrospray ionization (ESI), one for analysis by RP/UPLC-MS/MS using negative ion mode ESI, one for analysis by HILIC/UPLC-MS/MS using negative ion mode ESI, and one reserved for backup. Samples were placed briefly on a TurboVap^®^ (Zymark) to remove the organic solvent. The sample extracts were stored overnight under nitrogen before preparation for analysis (details on QA/QC are reported in the Supplementary Information).

#### Bioinformatics

The informatics system consists of four major components, the Laboratory Information Management System (LIMS, see Supplementary Information), the data extraction and peak-identification software, data processing tools for QC and compound identification, and a collection of statistical, visualization, and interpretation tools for use by data analysts. The hardware and software foundations for these informatics components are the LAN backbone and database servers running Oracle 10.2.0.1 Enterprise Edition.

#### Data extraction and compound identification

Raw data were extracted, peak-identified, and QC processed using hardware and software from Metabolon Inc. (München, Germany), built on a web service platform (Microsoft’s.NET technologies). Metabolon maintains a library based on authenticated standards that contains the retention time/index (RI), mass to charge ratio (*m*/*z*), and chromatographic data (including MS/MS spectral data) on all molecules present in the library. Biochemical identifications were based on: retention index within a narrow RI window of the proposed identification; accurate mass match to the library ±10 ppm; and the MS/MS forward and reverse scores. The use of all three data points was utilized to distinguish and differentiate potentially similar biochemicals. More than 4500 commercially available purified standard compounds have been acquired and registered into LIMS for analysis on all platforms. Additional mass spectral entries have been created for structurally unnamed biochemicals, which have been identified by virtue of their recurrent nature.

#### Metabolite quantification and block correction

Peaks were quantified as area-under-the-curve detector ion counts. For studies spanning multiple days, we performed a data adjustment step to correct block variation resulting from instrument inter-day tuning differences. Such adjustment was conducted by registering the daily medians to equal one, and adjusting each data point proportionately (block correction).

### Microbiota analyses

#### DNA extraction and sequencing

DNA was extracted from cecum content using a modified NucleoSpin^®^ Soil (Macherey-Nagel) protocol. The genomic DNA hitherto extracted was randomly sheared into fragments of ~350 base pairs (bp), which were used for library construction using NEBNext Ultra II Library Prep Kit for Illumina (New England Biolabs). These libraries were evaluated using Qubit 2.0 fluorometer quantitation and Agilent 2100 Bioanalyzer for the fragment size distribution. Quantitative real-time PCR was used to determine the concentration of the final library prior to sequencing. The library was sequenced using 2 × 150 bp paired-end sequencing on an Illumina HiSeq platform (see Supplementary Information for data preprocessing).

#### Mapping reads to gene catalog

High quality non-host reads were mapped to the Clinical Microbiomics in-house combined mouse fecal and cecum microbiome gene catalog using BWA mem (v. 0.7.16a) with options to increase accuracy (-r 1 -D 0.3). PCR/optical duplicates were removed using samtools (v. 1.6). For each individual read, the read was considered mapped if: an alignment of 100 bases, ≥95% identity in this alignment, and a mapping quality (MAPQ) ≥ 20. Reads meeting these criteria except for the MAPQ threshold were considered multimapped. Read-pairs were classified into one of three possible categories: unmapped, read-pairs in which both individual reads were unmapped; multimapped, read-pairs in which both individual reads were multimapped, or were mapped to genes in different metagenomics species (MGS), or one was multimapped and the other was unmapped; mapped to a gene, read-pairs in which both individual reads mapped to the same gene, or in which one read mapped to a gene and the other was unmapped, multimapped, or mapped to another gene in the same MGS (see Supplementary Information for their calculation and taxonomical annotation).

#### Functional annotation of gene catalog

Emapper software (v. 1.0.3, HMM mode) was used to compare each gene in the gene catalog to the EggNOG (v.4.5) orthologous groups database (http://eggnogdb.embl.de/), resulting in 4.47 M annotated genes (63%). These genes were then mapped from EggNOG to KEGG orthologies (KO) and modules (http://www.genome.jp/kegg) using MOCAT2 lookup tables (http://mocat.embl.de/), resulting in 3.26 M annotated genes (46%) assigned to a KO and 1.6 M genes (23%) assigned to a KEGG module.

#### Gene-level functional profiling of samples

We define gene proportion as the proportion of read-pairs mapped to each gene in the gene catalog, after removing unmapped or ambiguously mapped read-pairs. We define the functional abundance of a given EggNOG group as the total gene proportion for all genes belonging to the group.

### Brain Neu5Ac content

Neu5Ac in brain samples was quantified by HPLC-FLD. Deionized water (1 ml) was added to brain tissue samples kept in 2.0 ml Eppendorf safe-lock tubes (Eppendorf, Hamburg, Germany). These were immediately placed in an ice-water bath until homogenization (1 × 2 min, 30 Hz) in a Tissue Lyser II (Qiagen, Venlo, The Netherlands). Homogenized samples were quantitatively diluted with deionized water to obtain a slurry containing 1–2 mg/ml of brain tissue. To release bound Neu5Ac, 400 µl of slurry were transferred to 2 ml screw cap tubes (Corning, NY, USA) and formic acid (5.0 M, 400 µl) was added. The solution was placed then in a water bath (40 min, 100 °C), and thereafter cooled in an ice-water bath. Samples, containing a mixture of released and free Neu5Ac, were derivatized by quinoxaline formation as follows. Aliquots (200 µl) of hydrolysates were mixed with 200 µl of DMB stock solution (7 mM DMB, 18 mM sodium hydrosulfite, 750 mM 2-merpcaptoethanol and 1.3 M HAc) and incubated in a water bath (80 °C, 50 min). Derivatized solutions were diluted by addition of 400 µl of deionized water, mixed, centrifuged (10,000 × *g*, 5 min, 25 °C) and transferred to brown HPLC vials. In total, 10 µl were injected on to a Zorbax SB-Aq, 3.5 µm; 4.6 × 50 mm HPLC column. The elution profile was as follows: 0–2.5 min 100% MeOH:Water (25:75, 0.05% HAc), 2.6–3.6 min 100% MeOH: Water (80:20, 0.05% HAc), 3.7–7.0 min 100% MeOH:Water (25:75, 0.05% HAc). The resolved Neu5Ac and Neu5Gc peaks were integrated and quantified against a calibration curve mixture of Neu5Ac (15–150 ng/ml) and Neu5Gc (300–3000 ng/ml). A QC sample, consisting of a mice brain tissue pool (LNB 5362-021), was included in every analysis batch to assess the reproducibility of the method.

### Statistical analyses

The statistical models adopted in this study were set on the different parameters investigated. For all parameters investigated, the sample size was estimated using the free software G*Power 3.1 (https://www.psychologie.hhu.de/arbeitsgruppen/allgemeine-psychologie-und-arbeitspsychologie/gpower.html), chosen considering a power effect of 80%, an alpha error of 0.05. For behavioral and LTP data, statistical analyses—ANOVA for split-plot designs—were conducted using the software Statview 5.0 (Abacus Concepts, USA). The general model entailed a 2 individual genotype (WT vs. KO) × 2 maternal genotype (WT vs. KO) × *k* (repeated measurements, variable depending on the specific test). Individual and maternal genotype constituted between subjects factor and repeated measurements constituted within-subject factors. Fisher’s protected least-significance difference test was used for post hoc comparisons. Data are expressed as mean ± SEM. Statistical significance was set at *p* < 0.05. Finally, in those instances in which a given threshold was required to confirm that experimental subjects met the criterion for a given experimental paradigm, the observed phenotype was compared to the threshold through one-sample *t*-tests (the confidence interval was used to evaluate whether the criterion was achieved or not). For metabolomics, univariate statistical analyses were conducted using Welch’s two-sample *t*-tests and ANOVA on natural log-transformed data. Additional multivariate data analysis was conducted on the data using the software package SIMCA-P + (version 14.0, Umetrics AB, Umeå, Sweden). Principal component analysis was first employed to explore the variance within dataset and assessment of major confounders [45]. To identify metabolic differences, we employed partial least squares regression analysis, and its modification, orthogonal projection to latent structures [46]. For the microbiota analyses, conducted on 615 MGS and 259 KEGG modules, we used the linear mixed model (using the nlme P-package [47]) shown here:

$${\mathrm{LMM}} = \log \left( y \right)\sim {\mathrm{Dam}}_{{\mathrm{genotype}}} \times {\mathrm{Pup}}_{{\mathrm{genotype}}} + \left( {1|{\mathrm{Dam}}_{{\mathrm{ID}}}} \right).$$

This test was performed separately for pups at eye-opening and in adulthood, and post hoc analyses were corrected using Benjamini–Hochberg control for false-positive rate for multiple comparisons (cut off = 0.1).

## Results

### Maternal care and neonatal developmental milestones

WT and KO dams provided an indistinguishable level of active maternal care, assessed 2 h during the dark and 1 h during the light phase of the diurnal cycle (maternal genotype: *F*_1,16_ = 1.166, *p* = 0.296, Supplementary Fig. 2) [48]. Additionally, neither genetic nor lactational deprivation of 6′SL affected the maturation of gross morphology (body length and weight: offspring × maternal genotype: *F*_1,24_ = 0.699, *p* = 0.411 and *F*_1,36_ = 0.572, *p* = 0.457, respectively, Supplementary Fig. 3a, b) and onset of neurodevelopmental milestones (righting and grasping: offspring genotype × maternal genotype: *F*_1,36_ = 2.490, *p* = 0.128 and *F*_1,36_ = 0.267, *p* = 0.978, respectively, Supplementary Fig. 3c, d).

### General locomotion, sensorimotor gating, cognitive capabilities, and long-term potentiation (LTP)

While offspring genotype did not apparently influence general locomotion (offspring genotype: *F*_1,28_ = 0.218, *p* = 0.644), being reared to a KO dam (MILK and GENE + MILK mice) resulted in increased levels of general locomotion in adulthood (maternal genotype: *F*_1,28_ = 6.680, *p* = 0.015, Supplementary Fig. 4). Predictably, locomotor activity was lower during the light phase (18:00 to 6:00) and higher during the dark phase (6:00 to 18:00) (hours: *F*_23,644_ = 93.087; *p* < 0.0001).

**Fig. 2:**
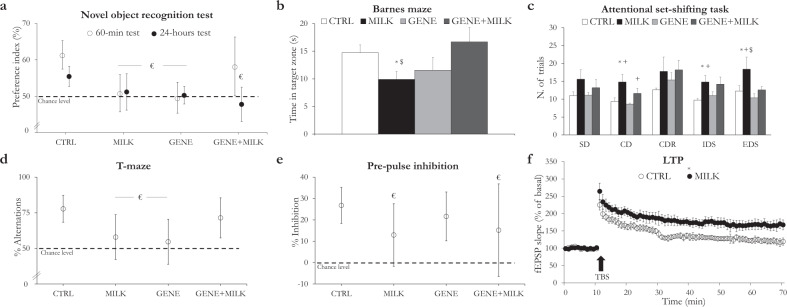
Behavioral and electrophysiological alterations in response to the absence of 6’SL from maternal milk and/or due to the deletion of the *St6Gal1* gene. Reduced access to 6′SL during lactation affects recognition memory (**a**), spatial reference memory (**b**), attentional capabilities (**c**), perseverative behavior (**d**), sensorimotor gating (**e**), and long-term potentiation (**f**). **a** Percent preference with 95% confidence intervals (CI, represented by whiskers) for the novel object during a 10-min test session performed 60min (open circles) and 24h (filled circles) after the habituation phase. The dashed line represents chance level; a CI intersecting the dashed line indicates that preference index was not statistically different from chance. In both test sessions, only CTRL subjects showed preference for the novel object compared to the familiar one. **b** Time spent in the target zone of the Barnes maze during the probe trial conducted 7 days after the last training session. Spatial memory retention varied between experimental groups (offspring genotype×maternal genotype: *F*_1,36_ = 6.120, *p* = 0.020, *N* = 10 per group). Specifically, CTRL mice spent more time in the target zone compared to MILK group (*p* = 0.023 in post hoc tests). **c** Number of trials to attain the criterion (eight correct choices out of ten consecutive trials) in different stages of the task: simple discrimination (SD); compound discrimination (CD); compound discrimination reversal (CDR); intra-dimensional shift (IDS); and extra-dimensional shift (EDS). While there is no significant difference between groups in the SD phase (offspring genotype: *F*_1,28_ = 0.529, *p* = 0.473; maternal genotype: *F*_1,28_ = 3.871, *p* = 0.059; offspring genotype×maternal genotype: *F*_1,28_ = 0.529, *p* = 0.473; CTRL: *N* = 8, MILK: *N* = 9, GENE: *N* = 8, GENE + MILK: *N* = 7) during CD, the number of trials needed to reach the criterion varied depending on the maternal genotype (offspring genotype: *F*_1,28_ = 0.759, *p* = 0.391; maternal genotype: *F*_1,28_ = 11.627, *p* = 0.002; offspring × maternal genotype: *F*_1,28_ = 0.007, *p* = 0.934; CTRL: *N* = 8, MILK: *N* = 9, GENE: *N* = 8, GENE + MILK: *N* = 7). Specifically, compared to CTRL and GENE, MILK mice required more trials to attain the criterion (*p* = 0.0218 and *p* = 0.0041, respectively, in post hoc tests). Additionally, compared to GENE group, GENE + MILK required higher number of trials to complete the phase (*p* = 0.023 in post hoc tests). During CDR phase, groups did not show significant difference (offspring genotype: *F*_1,28_ = 0.171, *p* = 0.682; maternal genotype: *F*_1,28_ = 3.212, *p* = 0.084; offspring × maternal genotype: *F*_1,28_ = 0.872, *p* = 0.358; CTRL: *N* = 8, MILK: *N* = 9, GENE: *N* = 8, GENE + MILK: *N* = 7). In IDs phase, the numbers of trial varied depending on maternal genotype (offspring genotype: *F*_1,28_ = 0.425, *p* = 0.520; maternal genotype: *F*_1,28_ = 7.892, *p* = 0.009; offspring × maternal genotype: *F*_1,28_ = 2.546, *p* = 0.122; CTRL: *N* = 8, MILK: *N* = 9, GENE: *N* = 8, GENE + MILK: *N* = 7). Specifically, compared to CTRL and GENE group, MILK group required more trials to attain the criterion (*p* = 0.003 and *p* = 0.017, respectively, in post hoc tests). Finally, experimental groups varied depending on both offspring and maternal genotype in number of trial needed to reach the criterion during the EDs phase (offspring genotype: *F*_1,28_ = 8.236, *p* = 0.007; maternal genotype: *F*_1,28_ = 6.461, *p* = 0.017; offspring × maternal genotype: *F*_1,28_ = 1.795, *p* = 0.191). Specifically, MILK group required more trials to attain the criterion compared to CTRL, GENE, GENE + MILK groups (*p* = 0.008, *p* = 0.0005, *p* = 0.006, respectively, in post hoc tests). Data are expressed as mean ± SEM; data on errors to criterion are reported in the Supplementary Information (see Supplementary Fig. 5). **d** Percentage of alternations (open circles) with 95% CI (whiskers) between the two arms of the T-maze. The dashed line represents chance level. CTRL subjects exhibited an intact natural tendency to alternate between the two arms of the maze (average 77.78%; 95% CI 68.19–87.36). **e** Percent inhibition of the startle reflex measured as PPI = [(A − B)/A × 100]: A is the Galvanic reflex in response to the startle stimulus alone, and B is the response to prepulse plus pulse stimuli (open circles) with 95% CI (whiskers). The dashed line represents chance level; a CI intersecting the dashed line indicates that the percent inhibition was not statistically different from chance and that mice failed to inhibit the startle reflex in response to the presentation of the prepulse. Mice of CTRL and GENE groups exhibited prepulse inhibition, while mice of MILK and GENE + MILK groups failed to exhibit it. **f** fEPSPs recorded in the CA1 area of hippocampal slices; long-term potentiation (LTP) was induced by theta-burst stimulation (TBS) of Schaffer collaterals and varied depending on the rearing dam (maternal genotype: *F*_1,14_ = 8.421, *p* = 0.012). MILK mice (*N* = 6 slices from three animals) showed an increased LTP compared to CTRL subjects (*p* = 0.04 in post hoc tests). The number of slices for each condition were: CTRL: *N* = 10 slices from five animals; MILK: *N* = 6 slices from three animals; GENE: *N* = 11 slices from five animals; GENE + MILK: *N* = 10 slices from five animals; data concerning the GENE vs. GENE + MILK comparison are reported in Supplementary Fig. 6. * indicates *p* value < 0.05 compared to CTRL group; + indicates *p* value < 0.05 compared to GENE group; $ indicates *p* value < 0.05 compared to GENE + MILK group; *p* values were calculated using analysis of variance (ANOVA). All bars represent mean ± SEM. € indicates that experimental group did not differ from chance.

Neonatal access to 6′SL-deficient maternal milk affected recognition and spatial reference memory, attentional capabilities, perseverative behavior, sensorimotor gating, and LTP in adulthood (Fig. 2a–f). Specifically, when requested to discriminate between a familiar and an unfamiliar object, CTRL subjects showed a preference for the unfamiliar one both 60 min and 24 h after familiarization (Fig. 2a). Such phenotype was never observed in MILK and GENE subjects, and was observed in GENE + MILK subjects only in the short-term but not in the long-term (Fig. 2a). Between group differences were not apparently related to variations in locomotion or anxiety whereby experimental subjects exhibited indistinguishable levels of distance moved or preference for the periphery of the open field during the 30-min pre-exposure session (see Supplementary information). MILK mice also exhibited a deficit in spatial reference memory (Fig. 2b), whereby, one week after the last training session, compared to CTRL, they showed a reduced capability to remember the location of a hole associated with the escape from an adverse situation. Such an impairment was not observed in GENE and GENE + MILK mice, albeit the former were also indistinguishable from MILK individuals. Similar to what observed in the NOR and T-maze, differences in reference memory were not dependent on general locomotion (see Supplementary information). To investigate potential alterations in executive functions, we conducted the attentional set-shifting task which evaluates stuck-in-set perseveration as a function of the ability to disregard a previously acquired rule in favor of a new one upon attainment of the learning criterion (Fig. 2c). In accordance with predictions, MILK mice showed increased perseveration across several stages of the test; albeit less pronounced, MILK + GENE mice showed an analogous impairment (see Fig. 2c and Supplementary Fig. 5). This increased perseveration in MILK mice was then confirmed in the T-maze test. Predictably, when faced with the possibility to access a previously explored arm or a new one in a binary choice paradigm, CTRL and GENE + MILK mice showed a robust preference for the novel arm. Such preference was absent in the other experimental groups thereby suggesting the presence of a perseverative behavior deficit due to the lack of 6′SL in early life diet or to the constitutive absence of the *St6Gal1* gene, but not when these factors were combined (Fig. 2d). These differences were not dependent upon general locomotion whereby subjects of all experimental groups required a similar latency to reach the chosen arm (see Supplementary information). To gain additional behavioral insights as to the function of the PFC (involved both in the attentional set-shifting and T-maze), we investigated PPI. Accordingly, MILK and GENE + MILK mice exhibited major impairments in PPI whereby they failed to show an inhibition beyond chance level in response to the prepulse stimulus (Fig. 2e). To investigate the fundamental mechanisms underlying the observed memory deficits, we addressed LTP in hippocampal slices. While basal synaptic transmission and paired-pulse facilitation of field excitatory postsynaptic potentials (fEPSPs) was comparable across experimental groups, LTP in CA1 was affected by maternal genotype. Specifically, while the stimulation protocol resulted in a long-lasting increase of fEPSP slope in CTRL mice (122.5 ± 13.35% of basal slope in the last 10 min of washout, *N* = 10 slices from five animals), such increase was higher in MILK mice (167.3 ± 16.49% of basal slope in the last 10 min of washout, *N* = 6 slices from three animals; *p* = 0.07 vs. CTRL group in post hoc test). Interestingly, in GENE + MILK mice the LTP magnitude (141.8 ± 8.12% of basal slope in the last 10 min of washout, *N* = 10 slices from five animals) was comparable to MILK group, but resulted higher than the potentiation found in GENE mice (123.6 ± 18.49% of basal slope in the last 10 min of washout, *N* = 11 slices from five animals; *p* = 0.06 vs. GENE + MILK group in post hoc test) (see Fig. 2f and Supplementary Fig. 6).

### Gene expression, metabolomics, and gut microbiota

**Fig. 3:**
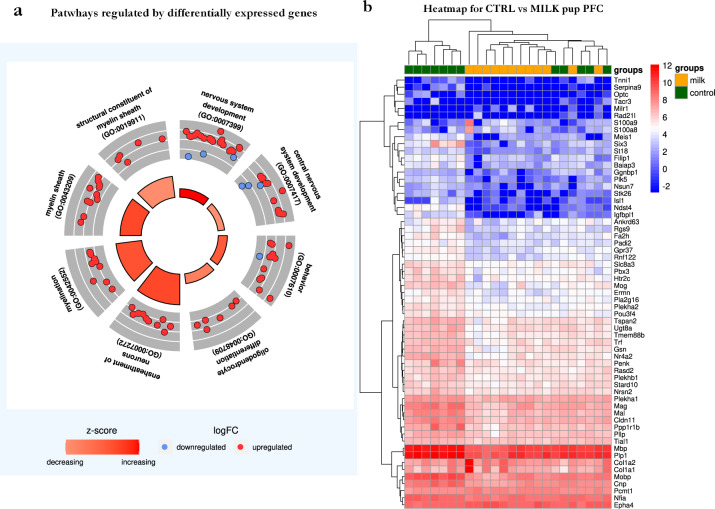
Gene expression alteration observed in response to the absence of 6′SL from maternal milk and/or due to the absence of the *St6Gal1* gene. **a** Circle plot presenting some of the pathways regulated by differentially expressed genes. The red boxes in the middle show the *z*-score, which is a normalized count of the number of up- or downregulated genes. The outer circle shows a scatter plot of the logFC for each of the assigned genes. The red and blue points represent over or under expression of the genes involved in each pathway. **b** Heatmap of 61 differentially expressed genes. Each cell represents the normalized log2 expression of each gene in each sample. Each row is a gene, each column is a sample. Dendrogram were obtained using hierarchical clustering with complete linkage on both features and samples. Samples are colored according to their group (milk or control) (Color figure online).

Data analysis suggests that the effects of 6′SL are mediated via modification in the expression of genes involved in brain development in the PFC (Fig. 3a, b). At eye-opening, MILK mice were characterized by a significant downregulation of 53 genes and a significant upregulation of eight genes compared to CTRL mice. Pathway analyses showed that downregulated genes are primarily involved in the formation and patterning of neuronal circuits (e.g., gangliosides or polySia-NCAM; Fig. 3a). Interestingly, several genes associated with myelination have also been demonstrated to be modulated in the MILK group. The alterations observed in the PFC at eye-opening appeared region- and age-specific. Specifically, gene expression analyses conducted in the PFC collected in adulthood resulted in a significant upregulation of two genes in the MILK group and in the downregulation of two genes and an upregulation of two genes in the GENE group. For the CA1/CA3 regions of the hippocampus, at eye-opening, we observed no changes in the GENE group and one significantly upregulated and one significantly downregulated gene in the MILK group. In adulthood, we observed two upregulated genes in the GENE group and six in the MILK group. For these ages and brain regions, such low numbers of differentially expressed genes did not allow us to identify specific pathways that could be impacted in these experimental groups either at eye-opening or in adulthood. These effects on gene expression were observed on a background of no significant differences between the four groups in terms of total Neu5Ac brain content (data not shown).

**Fig. 4:**
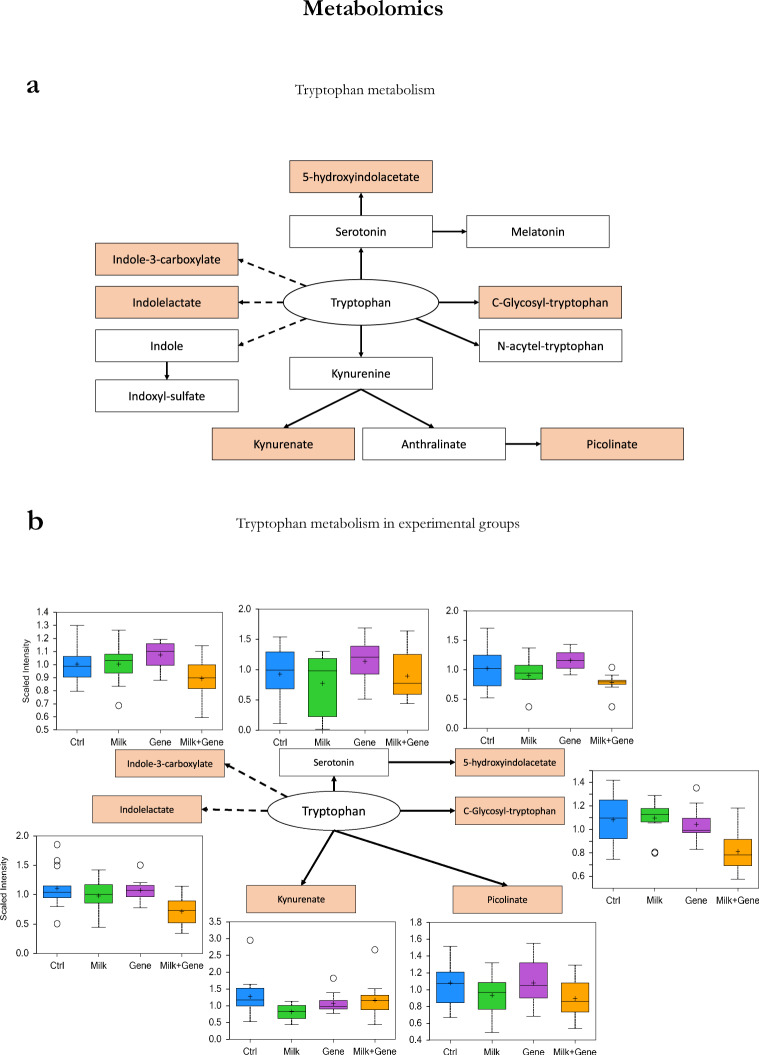
Alterations in tryptophan metabolism observed in response to the absence of 6’SL from maternal milk and/or due to the deletion of the *St6Gal1* gene. Reduced access to 6′SL during lactation perturbs the tryptophan metabolic pathways (**a**), and results in distinct circulating metabolite concentrations in blood (**b**). **a** Main metabolic intermediates in tryptophan metabolism. Solid lines represent endogenous mammalian metabolic relationships, dashed lines indicate host-microbial metabolic interactions. Red boxes indicate an interaction between group and metabolite levels at *p* value < 0.05 using analysis of variance (ANOVA). **b** Overview of blood circulating concentrations for statistically significant metabolites (red boxes). Serotonin was highlighted as an additional statistically significant metabolite using multivariate data analysis, and is displayed for information. All bars represent mean ± SEM (Color figure online).

Plasma metabolomics revealed that the tryptophan metabolism showed a profound remodeling in MILK and GENE+MILK pups, which were characterized by lower blood concentrations of serotonin, kynurenate, and picolinate (Fig. 4a, b). An additional indication that MILK mice exhibited an altered metabolism of tryptophan stems from host-microbial metabolism of indole molecules derived from tryptophan metabolism, such as indolelactate or indole-3-carboxylate. To gain further insight into this possibility, we investigated fecal microbiota composition. Microbiome metagenomics analysis did not show any significant difference in MGS richness (eye-opening, offspring genotype: *F*_1,45_ = 0.255, *p* = 0.62; maternal genotype: *F*_1,45_ = 3.7, *p* = 0.061; offspring × maternal genotype: *F*_1,45_ = 0.76, *p* = 0.39; adult age, offspring genotype: *F*_1,41_ = 0.03, *p* = 0.86; maternal genotype: *F*_1,41_ = 0.15, *p* = 0.71; offspring × maternal genotype: *F*_1,41_ = 0.001, *p* = 0.97) or diversity (eye-opening, offspring genotype: *F*_1,45_ = 0.58, *p* = 0.45; maternal genotype: *F*_1,45_ = 2.23, *p* = 1.14; offspring × maternal genotype: *F*_1,45_ = 0.55, *p* = 0.46; adult age, offspring genotype: *F*_1,41_ = 0.39, *p* = 0.54; maternal genotype: *F*_1,41_ = 2.13, *p* = 0.15; offspring × maternal genotype: *F*_1,41_ = 0.02, *p* = 0.89) either at eye-opening or at adult age.

**Fig. 5:**
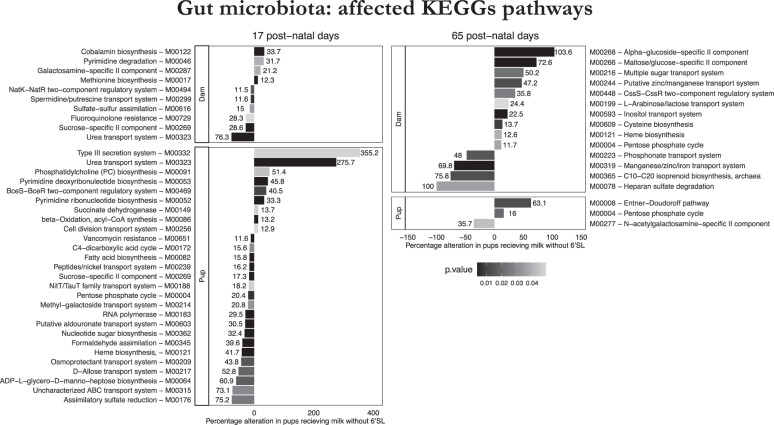
KEGGS pathway affected >10% compared to CTRL in metagenomics microbiota composition analyses at 17 postnatal days (left panels) and 65 postnatal days (right panels). Microbiota KEGGs pathway impacted by the milk absence of 6′SL or the deletion of *St6Gal1* gene. Grayscale coloring is providing indication of the *p* value and length of the bar indication of the percentage of increase (left) or decrease (right) of control group.

To evaluate the role of 6′SL on gut microbiota, we used a total of 615 MGS. At eye-opening, we observed a MILK effect for 30 MGS (0.0007 < *p* < 0.048), a GENE effect for 54 MGS (0.0001 < *p* < 0.048) and significant GENE + MILK interactions for 30 MGS (0.0012 < *p* < 0.045; see Supplementary Table 2). In adulthood, we observed a MILK effect for 28 MGS (0.0007 < *p* < 0.049), a GENE effect for 40 MGS (0.001 < *p* < 0.049) and significant GENE + MILK interactions for 29 MGS (0.006 < *p* < 0.049; see Supplementary Table 3). To understand the functional impact of the microbiota diversity, we investigated those KEGG pathways that showed a >10% significant change (abundance difference in experimental group relative to control group) using the same linear model as used for MGSs analysing an impact of either the MILK or the GENE group. This resulted in a total of 259 KEGG modules. At eye-opening, 19 modules were associated with an absence of 6′SL in maternal milk (0.004 < *p* < 0.049), while 41 with the genotype of the pup (0.003 < *p* < 0.049) and 17 with an interaction of both (0.002 < *p* < 0.048; see Supplementary Table 4). In adulthood, 16 modules were associated with MILK (0.002 < *p* < 0.049), 4 with GENE (0.02 < *p* < 0.04) and 6 with the interaction between MILK and GENE (0.005 < *p* < 0.04; see Fig. 5 and Supplementary Table 5). When focusing on the modules exhibiting a >10% significant change compared to CTRL, we observed at eye-opening ten modules altered in the MILK group and 27 modules altered in the GENE group (Fig. 5a). Interestingly, the number of modules altered in adulthood was higher in the MILK group, 14, and lower in the GENE group, 3 (Fig. 5b). Relative abundances of MGS and their representative microbiota at eye-opening and in adulthood are listed in Supplementary Tables 6 and  7, respectively.

## Discussion

Our data indicate that the absence of 6′SL in maternal milk during lactation results in long-lasting PFC-mediated impairments in executive functions and suggest that these effects are mediated via short-term alterations in the expression of genes responsible for the formation and patterning of neuronal circuits. Additionally, adopting a comprehensive approach, we observed that behavioral deficits are associated with alterations at the level of the serotoninergic system, as indicated by the analysis of plasma metabolomics and gut microbiota. Ultimately, our data confirm that 6′SL in maternal milk is selectively involved in the development of cognitive capabilities whereby it persistently adjusts the neuroanatomical and neurochemical pathways sustaining attention, memory, and perseverative behaviors.

While we did not quantify 6′SL milk content, empirical, and theoretical considerations indicate that KO dams provided milk devoid of 6′SL. From an experimental perspective, Fuhrer et al. [8] collected milk in *St6Gal1*^−/−^ KO lactating dams (the same used herein) and confirmed that the content of 6′SL was below detection threshold. From a theoretical perspective, there are only two existing genes potentially transferring sialic acid in 6′ position (thus, the only possible path to synthesize 6′SL): *St6Gal1* and *St6Gal2*. While *St6Gal1* gene undergoes a 15- to 20-fold increase in the mammary gland during the first and second postpartum week (respectively), *St6Gal2* gene increases its expression only during the second postpartum week and such an increase is only fivefold [8]. Additionally, *St6Gal2* gene expressed in bacteria failed to exhibit any activity toward oligosaccharides such as Galβ1,3GalNAc, Galβ1,3GlcNAc, lactose, and lacto-N-tetraose, an activity observed when bacteria were expressing the *St6Gal1* gene [49]. These findings strongly support the notion that 6′SL is not synthesized in *St6Gal1*^−/−^ KO mice.

With respect to memory, several authors consistently reported that the exogenous supplementation of sialylated oligosaccharides exerts beneficial effects in rats [19], piglets [18], and preterm pigs [20]. Yet, these studies did not allow univocal conclusions whereby dietary supplementation either occurred under conditions of physiological availability [18, 20] or lacked specificity [19]. Thus, whilst in [18, 20] sialic acid was administered to control subjects, Oliveros et al. [19] administered sialic acid to rats reared to dams that delivered their offspring 16 days before. Although this experimental procedure guaranteed that neonate rats were reared in a condition of poor 6′SL availability, it nonetheless lacked specificity whereby, compared to early-stage, a late-stage lactating dam provides a milk characterized by remarkable variations in nutrients which extend beyond sialic acid content. Leveraging a cross-fostering design, we overcame this limitation by selectively depriving developing offspring of 6′SL. Furthermore, to integrate behavioral findings with electrophysiological outcomes, we conducted an LTP study and observed that this parameter is altered in response to neonatal deprivation of 6′SL. This finding is in agreement with previous data collected in healthy piglets [18], preterm pigs [20], and rodents [19]. LTP studies have been conducted in temporal proximity with the behavioral tasks mapping onto the memory domain (i.e., between weeks 8 and 12). Specifically, LTP experiments were coincident with NOR (week 11, recognition memory) and T-maze (week 12, working memory), and proximate in time with PPI (week 13, not memory-related) and Barnes maze (week 14, spatial memory). This time window has also been selected based on our consolidated experience with the study of mouse hippocampal transmission and plasticity during this life stage [39, 50] and on the fact that, at this age, brain development is anatomically and biochemically complete [51, 52].

Beside memory deficits, our data indicate that the long-term regulatory role of 6′SL is not limited to memory but also extends to executive functions (attentional set-shifting [35] and perseverative behavior [31]). Deficits in the latter have been previously associated with alterations at the level of the prefrontal cortex [35, 53]. To further explore the functionality of this brain area, we evaluated sensorimotor gating through the PPI test [54, 55]. In accordance with the observed behavioral impairments, we showed that adult mice reared to dams providing 6′SL-deficent milk exhibited a complete loss of function, whereby they failed to inhibit their startle response above chance level.

To further detail the role of 6′SL at the level of the brain areas involved in the cognitive functions assessed, we performed an RNA-seq analysis on PFC and hippocampal samples collected at eye-opening (i.e., when maternal milk still constituted the only available food source) and in adulthood. These data support the hypothesis that the 6′SL-dependent long-term adjustments are mediated via short-term, PFC-specific alterations in gene expression. Thus, pathway analyses showed that, at eye-opening, MILK mice exhibited downregulation of genes directly involved in brain development. Additionally, we observed that the expression of several genes associated with myelination was altered in the MILK group. This suggests that not only is 6′SL necessary for establishing neuronal circuits, but also that it impacts myelination, possibly through a reduction in sialylated binding targets for the myelin associated glycoprotein [12, 13]. The anatomical and temporal selectivity of these effects was strengthened by the observation that gene expression differences were not observed in the hippocampus (neither at eye-opening nor in adulthood) and that PFC-specific differences nearly vanished in adulthood where only six genes exhibited altered expression in MILK mice, thus not allowing identification of specific pathways impacted. One caveat to this conclusion is related to the fact that the brain samples for gene expression analysis, in adulthood, have been collected in mice derived from two independent cohorts previously exposed to several behavioral tests. Thus, we cannot exclude that, had we conducted gene expression analyses in naïve subject, we may have observed a differential regulation of gene expression in the experimental groups. Yet, our decision to conduct these analyses in non-naïve individuals stems from the compromise between the extensive characterization of the study population and the need to minimize the invasiveness of the behavioral test battery and the total number of subjects utilized in the study (in accordance with the 3R’s principle [56]). In the light of this constrain, we opted to conduct postmortem analyses on a heterogenous population consisting of individuals performing different test batteries. It is important to emphasize that the samples analyzed were counterbalanced across groups per study cohort (i.e., each study cohort contributed an equal number of brain samples to each of the experimental groups in a balanced fashion). The scientific value of a heterogeneous study population analyzed through a random block design has been recently clarified by [57] and further discussed by [58].

To further investigate the regulatory role of 6′SL, we employed plasma metabolomics to measure the metabolic phenotypes of pups and define the influence of 6′SL on their endogenous metabolism and host-microbial metabolic interactions. Albeit indirect, these quantifications further support the selective role of 6′SL in the adjustment of executive functions. Specifically, we observed that maternal 6′SL deprivation remarkably influenced tryptophan metabolism. This phenotype may contribute to the deficits in behavioral flexibility [59] whereby: tryptophan represents the primary serotonin precursor [60]; and altered serotonin metabolism in the PFC has been associated with deficits in executive functions in humans and rodents [61]. Finally, the observed changes in the kynurenine pathways may also contribute to the observed alterations in cognitive development in the light of their regulatory role at the level of glutamatergic and cholinergic circuitry [62, 63].

To further detail the developmental mediators of the observed alterations, we investigated the gut microbiota in the light of its regulatory role on host physiology at the level of peripheral and CNS. Concerning the latter, species present in the gut synthesize not only metabolites, but also key neurosignalling molecules and their precursors (e.g., serotonin, catecholamine, tryptophan [23, 24]). Accordingly, we observed that lactational deprivation of 6′SL and/or altered gut glycosylation patterns (GENE) influenced the concentrations of specific microbes which have been already shown to modulate tryptophan metabolic pathways (serotonin, indoles, kynurenine) [64, 65].

When assessing the functional role of the altered MGSs, we observed that the influence of 6′SL deprivation was much more prominent in the short- then in the long-term, whereby the number of altered KEGG pathways was higher at eye-opening than in adulthood. Specifically, during the transition from milk to a solid diet we observed most pronounced alterations when 6′SL was missing in milk. These included increased abundance of the KEGG modules for urea transport and type III secretion system. The latter, used primarily by gram negative bacteria to deliver effectors into the host cells, is of special interest as it indicates a likely increased and presumably negative microbiota–host interaction with consequences for the microbiota and gut ecology as a whole due to host cell responses [66].

Additionally, notwithstanding a limitation related to the fact that our annotation referred to levels of taxonomy higher than the genus, we observed a lower abundance of Firmicutes, one of the phyla known to directly modulate serotonin synthesis [67] via an influence on tryptophan metabolism [65]. Accordingly, a previous study investigating the supplementation of 6′SL in 6–8-week-old mice [68] reported changes in diversity as well as an increase in Firmicutes and decrease in Bacteroidetes taxonomic groups. Thus, the observation that independent studies, conducted adopting complementary methodologies (deprivation vs. supplementation), led to similar outcomes suggests that 6′SL may alter tryptophan metabolism via an alteration in Firmicutes in gut microbiota [64].

While the core aim of our study was to investigate the role of 6′SL deprivation only during lactation, our experimental design also allowed us to isolate a potential impact of the *St6Gal1* gene deletion per se (GENE group). These mice exhibited a significant impairment in working memory. Regarding the mediating mechanisms, several lines of evidence indicate that *St6Gal1* is involved in maturation (somatic cell reprogramming) and in the structuring of immune functions (e.g., activation of B lymphocytes [25, 69, 70]), some of which have been reported to impact cognitive functions [71].

When analyzing gene expression, the number of genes altered was too small to allow the identification of specific pathways, suggesting that the *St6Gal1*^−/−^ KO resulted in a minimal impact on PFC or HPC gene expression either at eye-opening or in adulthood. When investigating plasma metabolite profiles in GENE mice, we observed only minimal changes which were not associated with identifiable pathways. This finding may suggest that the constitutive knock-out of the *St6Gal1* gene does impinge on plasma metabolomics.

However, when we looked at the microbiota functional capacity, we observed that at eye-opening the *St6Gal1* KO mice exhibited significant changes in 41 KEGG pathways. In adulthood, the total number of KEGG’s pathways altered were only 4, thus suggesting that the constitutive *St6Gal1* KO resulted in a very strong impact during the establishment of the gut microbiota early in life. Yet, such an effect appeared to be transient whereby, in adults, the GENE effect nearly vanished. This transient nature may be due to a key role of sialylation in the early microbiota establishment, in particular when weaning from the milk-based diet to the solid diet.

Ultimately, the constitutive deletion of *St6Gal1* resulted in more limited phenotypes compared to WT mice consuming milk devoid of 6′SL, and such a differential effect also extended to gut microbiota composition: specifically, while in the short-term both MILK and GENE mice exhibited substantial alterations in gut microbiota, these effects were nearly absent in adult GENE mice and still present in MILK individuals.

While both MILK and GENE groups exhibited deficits in cognitive capabilities, GENE + MILK mice were indistinguishable from CTRL individuals with respect to their behavioral phenotype. We propose a programming hypothesis. *St6Gal1* KO offspring present reduced gut sialylation leading to the observed altered gut microbiota. Similarly, animals receiving milk without 6′SL show alterations in the gut microbiota. Presumably, these alterations are directed by the specific missing sialylated components in the milk or the host, with the host immune system acting as an additional driver. These two driving forces may act in opposite directions in the MILK (no exogenous 6′SL) and GENE (no endogenous 6′-sialylation) groups, as we may conclude from the differences in the short- vs. long-term microbiota changes that we observed in GENE and MILK, respectively. This suggested opposite driving forces of the gut microbiota by genotype vs. nutrition results in a maladapted microbiota host pairing with consequent negative impact on brain functions. In the MILK + GENE group on the other hand, both the host glycosylation and immune responses and the nutrition may drive the microbiota host interaction in the same direction, hence not leading to incompatibility and maladaptation. Interestingly, several indole metabolites and glycosyltryptophan in plasma were observed to be different in the GENE + MILK group compared to control, MILK or GENE group, respectively. Albeit highly speculative, this may indicate that the presumed compensatory mechanisms underlying the lack of phenotypic alterations in GENE + MILK mice were not dependent on an alleged normalization, but rather involved different mechanism exerting opposite forces. Future studies are needed to clarify these aspects.

While our study indicates that the milk content of 6′SL exerts a profound impact on cognitive development, we cannot exclude the possibility that other maternal factors may also contribute to the phenotype observed in the progeny. Herein, to partly account for these factors, we extensively addressed maternal care through a detailed ethogram [27] and did not observe any difference between WT and KO lactating dams. Yet, maternal influences on individual phenotype extend beyond maternal care and include, for example, endocrine factors [72]. In the present study, to keep the disturbance of the nest to a bare minimum, we decided not to include endocrine measurements which would have implied a considerable degree of invasiveness. Future studies, specifically targeted toward a thorough phenotyping of KO dams, are therefore needed to further clarify this aspect.

In summary, our findings show that 6′SL in maternal milk is crucial for the development of cognitive capabilities and that limited access to this sialylated HMO early in life may persistently impair individual phenotypes in adulthood. This suggests that sialylated HMOs could be an important nutritional target in attempting to reduce the discrepancy between maternal and formula milk for brain and cognitive benefits. Further clinical studies and efficacy data are desirable to further the understanding of the role of HMOs in human brain and cognitive development.

## Supplementary information

Supplementary information

Supplementary figure 1

Supplementary figure 2

Supplementary figure 3

Supplementary figure 4

Supplementary figure 5

Supplementary figure 6

Supplementary table 2

Supplementary table 3

Supplementary table 4

Supplementary table 5

Supplementary table 6

Supplementary table 7
