# Supplementary material for: Sialylated human milk oligosaccharides program cognitive development through a non-genomic transmission mode
Source: Mol Psychiatry. 2021 Mar 4;26(7):2854–71. doi: 10.1038/s41380-021-01054-9 (PMC8505264; doi:10.1038/s41380-021-01054-9)
Supplement: Supplementary file 1 — Supplementary information [file 41380_2021_1054_MOESM1_ESM.docx]

**SUPPLEMENTARY INFORMATION**

**MATERIALS AND METHODS**

**Genotyping procedures**

To isolate genomic DNA, 0.5 to 0.3 cm of mouse tail biopsy samples were incubated at 55°C overnight in a thermoblock with gentle agitation (300 rpm) in 0.5-0.25 mL of Lysis Buffer (100mM Tri-HCl pH 8, 0.5% TWEEN® 20, 0.5% NP-40) with Proteinase K (ThermoFisher scientific) added to 0.1 mg/ml final concentration. After that, proteinase K inactivation was performed incubating samples at 75°C for 20 minutes. 2 ul of each mouse tail lysate was used as PCR template to perform a PCR reaction with GoTaq® DNA Polymerase (Promega) (0.65U GoTaq® DNA Polymerase (Promega) 1,5 mM MgCl2, 0,5 mM each dNTP, 0,6 uM primers, molecular biology grade water up to final volume of 25 uL) by using primers 15318 (Fw) 5’-ACTGTGGGGCTAACCTTTGA-3’ and 15319 (Rev) 5’-TGCACCATGACTCAGCTTCT-3’, as per B6.129-St6gal1tm2jxm/J strain genotyping protocol (https://www2.jax.org/protocolsdb/f?p=116:5:0::NO:5:P5_MASTER_PROTOCOL_ID,P5_JRS_CODE:9131,006901). Reactions were carried out using the following cycling conditions: 2 min at 95°C followed by 33 cycles of 95°C for 30 sec, 55°C for 30 sec and 72°C for 30 sec. A final extension step at 72°C for 5 min was performed after the cycles. After this step, PCR reactions were kept at 4°C until being electrophoresed in 2% agarose 1×TBE (89 mM Tris base pH8, 89 mM boric acid and 2 mM ethylendiaminetetraacetic acid) gels. For visualization of electrophoresed PCR products, gels were stained with Ethidium bromide (0,006%) and digital images were captured in a CHEMIDOC MP Imaging System (Bio-Rad). Homozygous wild type, heterozygous mutant and homozygous mutant genotypes have been distinguished by their different band patterns (≈287 bp, ≈287 bp and 403bp, ≈403 bp respectively).

**Attentional set-shifting task**

On the day before testing, mice were given access to the apparatus for 30 min. Following this preliminary exposure, mice were trained to dig into food-baited bowls during a series of 9 consecutive trials. During the first three trials mice were allowed to explore the apparatus until two food rewards, both located on the surface of the empty bowls, were retrieved. Between trials 3-6, food rewards were located on the surface of the digging media inside the bowls and mice were allowed to explore the apparatus until retrieval. Between trials 6-9, food rewards were located underneath the digging media. This procedure allowed mice to perform reliable digging. A trial was initiated by raising the sliding wall to give the mouse access to the two digging bowls, only one of which was baited. Food-restricted mice (90% of their original body weight at the beginning of the experiment) were required to dig into a rewarded (food-baited) bowl to obtain highly palatable food pellets. Digging bowls varied across two dimensions (digging medium and scent). Digging media and odours exemplars used in this study are reported in Supplementary Table 1. The test consisted of the following stages: simple discrimination (SD); compound discrimination (CD); CD reversal (CDR); intra-dimensional shift (IDs); and extra-dimensional shift (EDs). During SD mice had to learn to discriminate between two different odours. In CD, the baited stimulus of the previous stage was presented together with another, newly introduced, irrelevant stimulus of the other dimension (digging medium). Despite the presence of the new stimulus, the correct and incorrect exemplars remained constant (e.g. cinnamon odour was rewarded when presented in combination with either sawdust or shredded paper while thyme was not rewarded independently of the digging medium, see **Supplementary Table 1**). At the end of this stage, mice had to perform CD reversal learning (CDR). For the reversal, the exemplars and the relevant dimension were unchanged: the mouse had to learn that the previously correct stimulus was now incorrect. In intra-dimensional shift (IDs), we changed all the stimuli, but odour remained the relevant one, while in extra-dimensional shift (EDs) digging medium became the food rewarded associated stimulus. A stage was considered complete when the mouse achieved a criterion of 8 correct trials out of 10. A session would continue until the animal ceased responding. Normally, mice would give a good profile of responses for about two hours, time after which they would just ignore the reward. Since the end of a session depended on the individual motivation to perform the task, subjects performed a variable number of trials each day.

| **Dimension** | **Pairing (exemplar 1)** | **Pairing (exemplar 2)** |
| --- | --- | --- |
| Odour | Cinnamon – Thyme | Anise - Thyme |
| Medium | Sawdust – Cotton | Sawdust – Paper chip |

**Supplementary Table 1.** Stimulus exemplars used in the task. Compound discriminations were based on fixed combinations of pairs of exemplars. The sequence of these combinations was presented in random combination.

**Gene expression**

*Libraries QC and sequencing*

Libraries are quantified with Quant it Picogreen (Life Technologies). Size pattern is controlled with the High Sensitivity NGS Fragment Analysis kit on a Fragment Analyzer (Agilent). Libraries are pooled at an equimolar ratio (i.e. an equal quantity of each sample library) and clustered at a concentration of 9 pM on single read (SR) sequencing flow cell (Illumina). Sequencing is performed for 65 cycles on a HiSeq 2500 (Illumina) using the HiSeq SR Cluster Kit v4 cBot, HiSeq SBS Kit V4 50 cycle kit (Sequencing by Synthesis). Primary data quality control is performed during the sequencing run to ensure the optimal flow cell loading (cluster density) and check the quality metrics of the sequencing run (QC30). The optimal number of density of clusters detected by image analysis is between 850 and 1000 K/mm2. Performing a run at optimal cluster density involves finding a balance between under clustering that maintains data of good quality but results in lower data output and over clustering that can lead to poor run performance. The percentage ≥Q30, means the percentage of bases with a quality Phred score of 30 or higher. It is a measure of the quality of the identification of the nucleobases generated. The Phred score is logarithmically linked to error probabilities. A Phred score equal to 30 means a probability of incorrect base assignment of 1 in 1000, so a base call accuracy of 99.9%. Regarding Illumina specification, the score should be a minimum of 80%.

Additional quality controls for the sequencing include:

- Total number of reads per samples with a target at 5 million reads
- The % of reads mapping onto the reference genome
- The % of reads corresponding to rRNA
- The % of PCR duplicates. For blood samples, the % of globin mRNA

**Metabolomics**

*QA/QC*

Several types of quality control samples are analysed in concert with the experimental samples. These include: 1) technical replicate samples derived from a pool of well-characterized human plasma (MTRX) or, alternatively, generated by combining a small portion of each (non-plasma) experimental sample (CMTRX), spaced evenly among experimental samples; 2) extracted water samples (process blanks) and solvent blanks; and 3) a cocktail of QC standards, carefully chosen not to interfere with the measurement of endogenous compounds, spiked into every analyzed sample, allowing instrument performance monitoring and aiding with chromatographic alignment. Tables 1 and 2 describe these QC samples and standards. Instrument variability is determined by calculating the median relative standard deviation (RSD) for the standards that were added to each sample prior to injection into the mass spectrometers. Overall process variability is determined by calculating the median RSD for all endogenous metabolites (i.e., non-instrument standards) present in each of the pooled MTRX (or CMTRX) technical replicate samples. Experimental samples are randomized across the platform run, with QC samples spaced evenly among the injections.

*Ultrahigh Performance Liquid Chromatography-Tandem Mass Spectroscopy (UPLC-MS/MS)*

All methods utilize a Waters ACQUITY ultra-performance liquid chromatography (UPLC) and a Thermo Scientific Q-Exactive high resolution/accurate mass spectrometer interfaced with a heated electrospray ionization (HESI-II) source and Orbitrap mass analyzer operated at 35,000 mass resolution. The sample extract is dried then reconstituted in solvents compatible to each of the four methods. Each reconstitution solvent contains a series of standards at fixed concentrations to ensure injection and chromatographic consistency. One aliquot is analysed using acidic positive ion conditions, chromatographically optimized for more hydrophilic compounds. In this method, the extract is gradient-eluted from a C18 column (Waters UPLC BEH C18-2.1×100 mm, 1.7 µm) using water and methanol, containing 0.05% perfluoropentanoic acid (PFPA) and 0.1% formic acid (FA). A second aliquot is also analysed using acidic positive ion conditions, but is chromatographically optimized for more hydrophobic compounds. In this method, the extract is gradient eluted from the aforementioned C18 column using methanol, acetonitrile, water, 0.05% PFPA and 0.01% FA, and is operated at an overall higher organic content. A third aliquot is analysed using basic negative ion optimized conditions using a separate dedicated C18 column. The basic extracts are gradient-eluted from the column using methanol and water, however with 6.5mM Ammonium Bicarbonate at pH 8. The fourth aliquot is analyzed via negative ionization following elution from a HILIC column (Waters UPLC BEH Amide 2.1×150 mm, 1.7 µm) using a gradient consisting of water and acetonitrile with 10mM Ammonium Formate, pH 10.8. The MS analysis alternates between MS and data-dependent MSn scans using dynamic exclusion. The scan range varies slightly between methods, but covers approximately 70-1000 m/z.

*LIMS*

The purpose of the Metabolon LIMS system is to enable fully auditable laboratory automation through a secure, easy to use, and highly specialized system. The scope of the Metabolon LIMS system encompasses sample accessioning, sample preparation, instrumental analysis and reporting, and advanced data analysis. All the subsequent software systems are grounded in the LIMS data structures, which has been modified to leverage and interface with the in-house information extraction and data visualization systems, as well as third party instrumentation and data analysis software.

*Curation*

A variety of curation procedures are performed to ensure that a high quality data set is made available for statistical analysis and data interpretation. The QC and curation processes are designed to ensure accurate and consistent identification of true chemical entities, and to remove those representing system artifacts, mis-assignments, redundancy, and background noise. Metabolon data analysts use internally-developed visualization and interpretation software to confirm the consistency of peak identification among the various samples. Library matches for each compound are checked for each sample and corrected if necessary.

**Microbiota analyses**

*Data preprocessing*

The raw FASTQ files were quality controlled using KneadData (v. 0.6.1) to remove low-quality bases and reads derived from the host genome as follows: Using Trimmomatic (v. 0.36), the reads were quality trimmed by removing leading or trailing bases with a Phred score below 20, and trailing bases in which the Phred score over a window of size 4 drops below 20. Trimmed reads shorter than 100 bases were discarded. Reads that mapped to the mouse reference genome GRCm38 (with Bowtie2 v. 0.2.3.2) were discarded. Read-pairs in which both reads survived filtering were retained; these were classified as HQNH reads.

*MGS relative abundance calculation*

For each MGS, the “core” genes were defined as the 100 genes specific for the MGS and with the highest correlation to the mean and lowest absolute deviation from the mean. An MGS counts table was created based on the total gene counts for the 100 core genes of each MGS. However, an MGS was considered detected only if read pairs were mapped to at least three of its 100 core genes; counts for MGSs that did not satisfy this criterion were set to zero. This MGS counts table was normalized according to effective gene length (accounting for read length) and then normalized to sum to 100%, resulting in relative abundance estimates of each MGS. Richness and diversity measured were based on downsampled (rarefied) MGS abundance profiles to control for uneven sampling. These were calculated by performing the above procedure on a downsampled gene counts table (generated by random sampling, without replacement, of HQNH read-pairs).

*Taxonomical annotation of MGSs*

MGSs were annotated by blasting catalog genes to the NCBI RefSeq genome database using various similarity thresholds to annotate at the different taxonomic levels (ranging from 65% for phylum to 95% for species) and requiring a minimum of 80% sequence coverage. We calculated the percentage of genes of each MGS that mapped to each species and assigned species level taxonomy to an MGS if >75% of its genes could be annotated to a given species. For genus, family, order, class, and phylum, we used 60%, 50%, 40%, 30% and 25% consistency respectively. Furthermore, at species and at genus level we allowed a maximum of 10% of the genes belonging to an MGS to be annotated to an alternative taxon.

**RESULTS**

*Object recognition memory test (ORMT, week 11)*

To control for potential differences in baseline levels of locomotion, we quantified the distance moved by each individual throughout the 30-min pre-exposure session and observed that the four groups did not differ in this parameter (maternal genotype: *F*_1,35_ = 2.399, P = 0.130; offspring genotype: *F*_1,35_ = 2.261, P = 0.142; offspring genotype × maternal genotype: *F*_1,35_ = 0.927, P = 0.342). To evaluate anxiety-related responses, we also analysed the percent time spent in the periphery of the open field and observed that, while all subjects strongly preferred this area compared to the central one, such a preference was indistinguishable across groups (maternal genotype: F_1,35_ = 0.188, P = 0.668; offspring genotype: F_1,35_ = 0.519, P = 0.476; offspring genotype × maternal genotype: F_1,35_ = 0.240, P = 0.627). Specifically, the average percent preference was: CTRL = 66.2 ± 1.1; MILK = 68.5 ± 2.4; GENE = 68.1 ± 2.2; GENE + MILK = 68.7 ± 2.3. During testing, experimental subjects exhibited indistinguishable levels of total object exploration, computed as the time spent exploring the novel object plus time spent exploring the familiar object (maternal genotype: F_1,35_ = 0.302, P = 0.586; offspring genotype: F_1,35_ = 0.054, P = 0.817; offspring genotype × maternal genotype: F_1,35_ = 0.248, P = 0.622). Specifically, the average time spent exploring both objects (s) was: CTRL = 308.2 ± 18.1; MILK = 292.1 ± 12.2; GENE = 304.1 ± 16.6; GENE + MILK = 303.3 ± 11.7.

*T-maze (week 12)*

To indirectly evaluate general locomotion in the T-maze, we measured the latency to reach the chosen arm by each individual in the light of the fact that such distance was highly comparable for all subjects. The analysis of this parameter suggested that general locomotion in the T-maze did not differ across experimental groups (maternal genotype: F_1,35_ = 0.150, P = 0.701; offspring genotype: F_1,35_ = 2.958, P = 0.094; offspring genotype × maternal genotype: F_1,35_ = 1.336, P = 0.255).

*Barnes maze (week 14)*

To control for differences in general locomotion, we evaluated the total distance moved on the experimental platform during the 90s probe trial. Data analysis revealed that while neither maternal nor offspring genotype *per se* affected general locomotion (maternal genotype: F_1,36_ = 0.061, P = 0.806; offspring genotype: F_1,36_ = 0.527, P = 0.473), being reared to a dam of a different genotype resulted in variations in this parameter (offspring genotype × maternal genotype: F_1,36_ = 5.347, P = 0.027). Specifically, we observed that, in the absence of differences in the other comparisons, GENE mice exhibited lower levels of locomotion compared to CTRL (p < 0.05 in post-hoc comparisons). The fact that such difference in locomotion was not paralleled by a difference in reference memory indirectly supports the view that the latter is not necessarily a function of the former in this test.

*Attentional set-shifting task (weeks 18-23)*

In accordance with previous studies, the CDR resulted the most difficult stage, whereby experimental subjects required a higher number of trials and committed more errors to reach the learning criterion (stage: *F*_4,122_ = 10.361; P = 0.0001, see **Supplementary Fig. 5**). Compared to offspring reared to WT dams, subjects reared to KO dams committed more errors to attain the criterion in several stages of the task (maternal genotype: *F*_1,28_ = 9.054, P = 0.005). In particular, MILK mice committed an overall higher number of errors compared to CTRL and GENE mice (respectively P = 0.010 and P = 0.007). During SD phase, MILK mice committed more errors than CTRL and GENE subjects (maternal genotype: *F*_1,28_ = 5.602, P = 0.025; P = 0.023 and P = 0.043 in post-hoc tests, respectively). Likewise, during the CD phase, MILK mice committed more errors than CTRL and GENE animals (maternal genotype: *F*_1,28_ = 15.959, P = 0.0004; P = 0.019 and P = 0.003 in post-hoc tests, respectively), and GENE+MILK subjects committed more errors than CTRL and GENE mice (respectively P = 0.019 and P = 0.004 in post-hoc tests). Finally, during the IDS phase, MILK mice committed more errors than CTRL and GENE animals (maternal genotype: *F*_1,28_ = 5.566, P = 0.025; P = 0.005 and 0.017 in post-hoc tests, respectively).

*Electrophysiology experiments*

fEPSPs were recorded in the CA1 area of hippocampal slices; LTP was induced by TBS of Schaffer collaterals. MILK mice showed an increased LTP compared to CTRL (P = 0.07 vs. CTRL group, Mann-Whitney test, see **Fig. 2f**). In GENE+MILK mice the LTP was comparable to MILK group but higher than that found in GENE mice (P = 0.06 vs. GENE+MILK group, Mann-Whitney test, see **Supplementary Fig. 6**). The number of slices for each condition was as follows: CTRL mice, n = 10 slices from 5 animals; MILK mice (n = 6 slices from 3 animals); GENE+MILK mice, n = 10 slices from 5 animals; GENE mice, n = 11 slices from 5 animals).

*Gut microbiota*

When analysing MGS, we found that, at eye opening (see **Supplementary Table 6** for average values in each experimental group and **Supplementary Table 2** for p values), 3 MGSs, MGS0652 (Clostridia sp.), MGS0364 (Lachnospiraceae sp.), and MGS0339 (Clostridiales sp.), showed significantly higher concentration in pups receiving milk with 6’SL (CTRL or GENE groups), and MGS0426 (Oscillospiraceae sp.) showed significantly higher abundances in pups receiving milk without 6’SL (MILK or GENE+MILK groups). Five MGSs, MGS0822 (Firmicutes sp.), MGS0066 (Lachnospiraceae sp.), MGS0460 (Bacteroidales sp.), MGS0489 (Clostridiales sp.), and MGS0221 (Lachnospiraceae sp.), showed significantly higher abundances in KO pups (GENE or GENE+MILK groups), while two MGSs, MGS0382 (Rikenellaceae sp.) and MGS0063 (Bacteroidetes sp.), showed significantly higher abundances in WT pups (CTRL or MILK groups). In adulthood (see **Supplementary Table 7** for average values in each experimental group and **Supplementary Table 3** for p values), 2 MGSs, MGS0428 (Clostridiales sp.) and MGS0194 (Clostridiales sp.), showed significantly higher abundance in response to absence of 6’SL from the milk (MILK and GENE+MILK groups). Three MGSs, MGS0657 (Oscillospiraceae sp.), MGS0390 (Clostridiales sp.), MGS0586 (Clostridiales sp.) showed significantly higher abundances in WT pups (CTRL and MILK groups).

**SUPPLEMENTARY FIGURE LEGENDS**

**Supplementary figure 1.** Libraries preparation workflow and principle.

Supplementary figure 2. Active maternal care: maternal care was assessed, during the first 10 days of life, through focal sampling over three one-hour periods (two during the dark phase, at 9:00 and 14:00, and one during the light phase, at 18:00). In each one-hour period, dams were observed 20 times at regular 3-min intervals. WT (dashed line, n = 8) and KO (solid line, n = 10). Data are expressed as average hourly frequency of active nursing (high/low/partial kyphosis and licking/grooming).

Supplementary figure 3. Neurodevelopmental milestones: maturation of developmental milestones in newborn mice born to WT or KO dams and in-fostered to dams of the same genotype or cross-fostered to dams of the different genotype. (a) Body length on PND3 and 7; (b) body weight gain between PND3 and PND 7; (c) latency (s) to acquire the upright position when the subject is placed on its back (righting reflex) measured on PND3 and 7; (d) strength of grasping measured as the angle at which experimental subject fall from a wire mesh grid which is slowly rotated from 0 to 180 degrees.

**Supplementary figure 4.** General locomotion: data are expressed as mean ± SEM; * indicates P-value < 0.05 compared to CTRL group; + indicates P-value < 0.05 compared to GENE group.

Supplementary figure 5. Attentional set-shifting capabilities: errors to attain the criterion (8 corrects trials out of 10) in different stages of the task: simple discrimination (SD); compound discrimination (CD); compound discrimination reversal (CDR); intra-dimensional shift (IDS); and extra-dimensional shift (EDS). * indicates P-value < 0.05 compared to CTRL group. + indicates P-value < 0.05 compared to GENE group. Data are expressed as mean ± SEM.

**Supplementary figure 6.** fESPS slope: data recorded in CA1 area of GENE and GENE+MILK groups after a theta-burst stimulation (TBS). + indicates P-value < 0.05 compared to GENE group. Data are mean ± S.E.M.

**Supplementary table 2.** List of P-values for abundances of metagenomics species (MGS) and their representative microbiota species at eye opening. All groups are compared with CTRL group. In bold P-value < 0.05.

**Supplementary table 3.** List of P-values for abundances of metagenomics species (MGS) and their representative microbiota in adulthood. All groups are compared with CTRL group. In bold P-value < 0.05.

**Supplementary table 4.** List of P-values for abundances of KEGGs module (Kyoto Encyclopedia of Genes and Genomes) at eye opening and their relative function and category. All groups are compared with CTRL group. In bold P-value < 0.05.

**Supplementary table 5.** List of P-values for abundances of KEGGs module (Kyoto Encyclopedia of Genes and Genomes) in adulthood and their relative function and category. All groups are compared with CTRL group. In bold P-value < 0.05.

**Supplementary table 6.** Relative abundances of metagenomics species (MGS) and their representative microbiota at eye opening.

**Supplementary table 7.** Relative abundances of metagenomics species (MGS) and their representative microbiota in adulthood.
