# Supplementary figures and images for: Sialylated human milk oligosaccharides program cognitive development through a non-genomic transmission mode
Source: Mol Psychiatry. 2021 Mar 4;26(7):2854–71. doi: 10.1038/s41380-021-01054-9 (PMC8505264; doi:10.1038/s41380-021-01054-9)

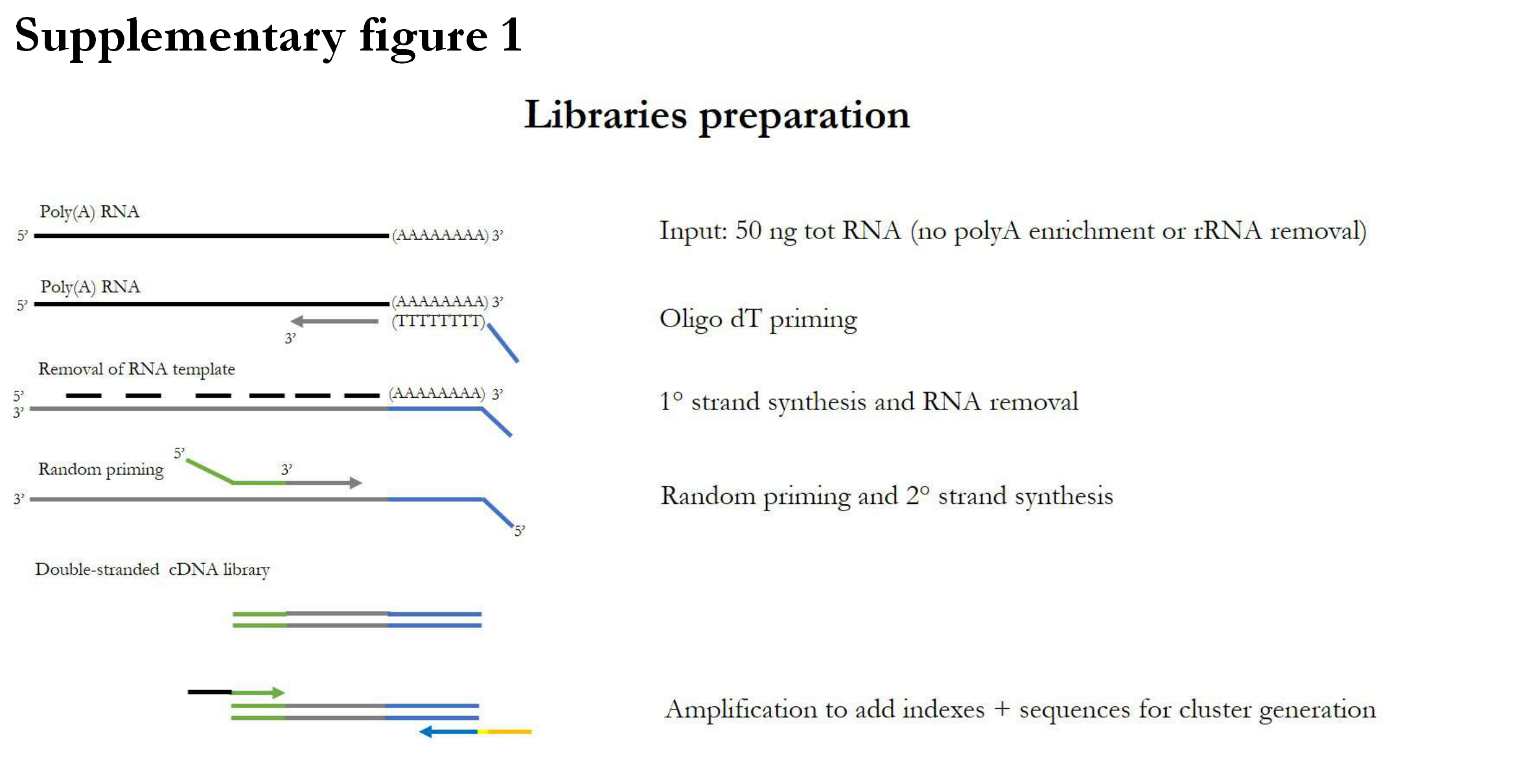

Supplement: Supplementary file 2 — Supplementary figure 1 [file 41380_2021_1054_MOESM2_ESM.tif]

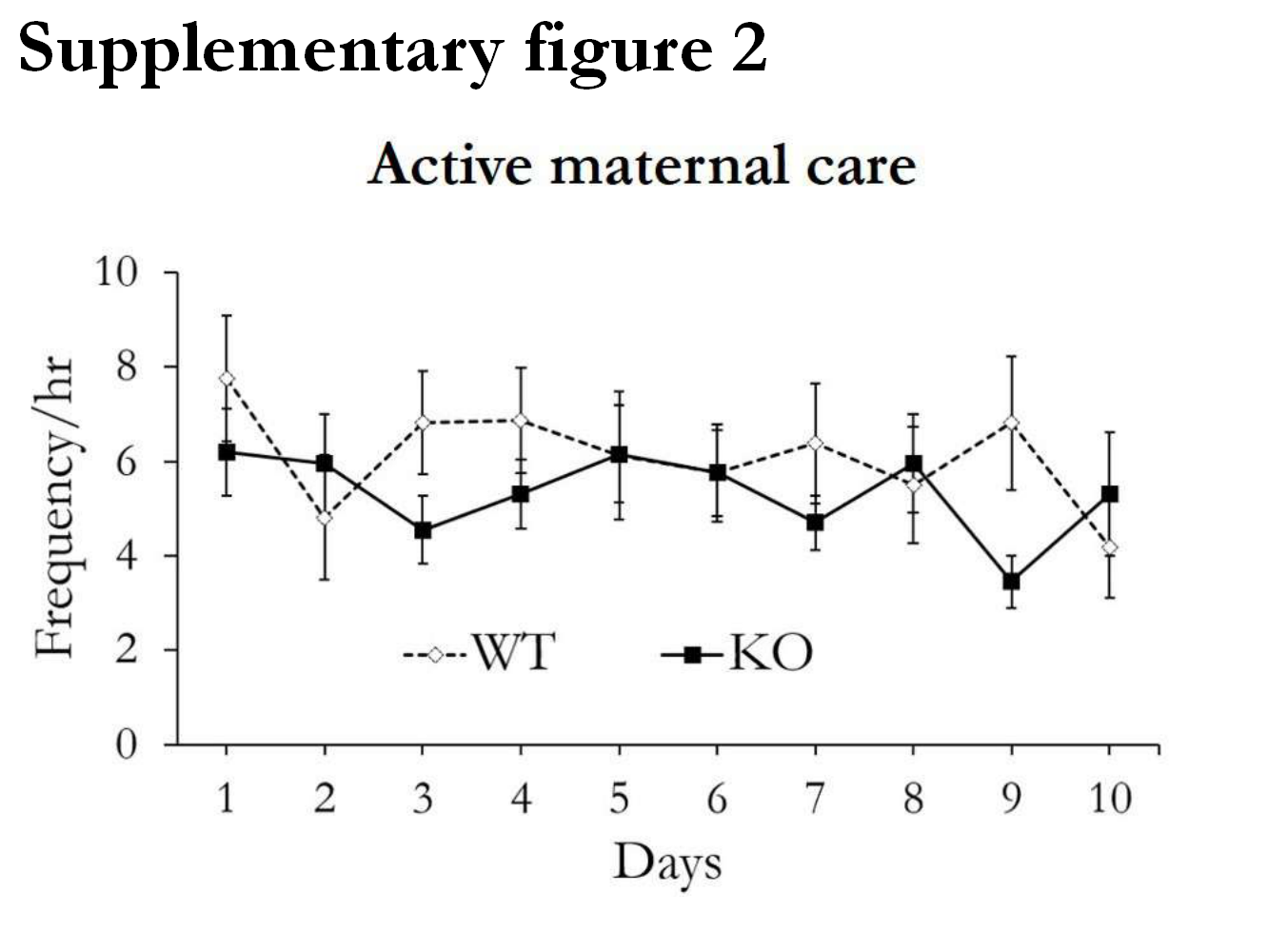

Supplement: Supplementary file 3 — Supplementary figure 2 [file 41380_2021_1054_MOESM3_ESM.tif]

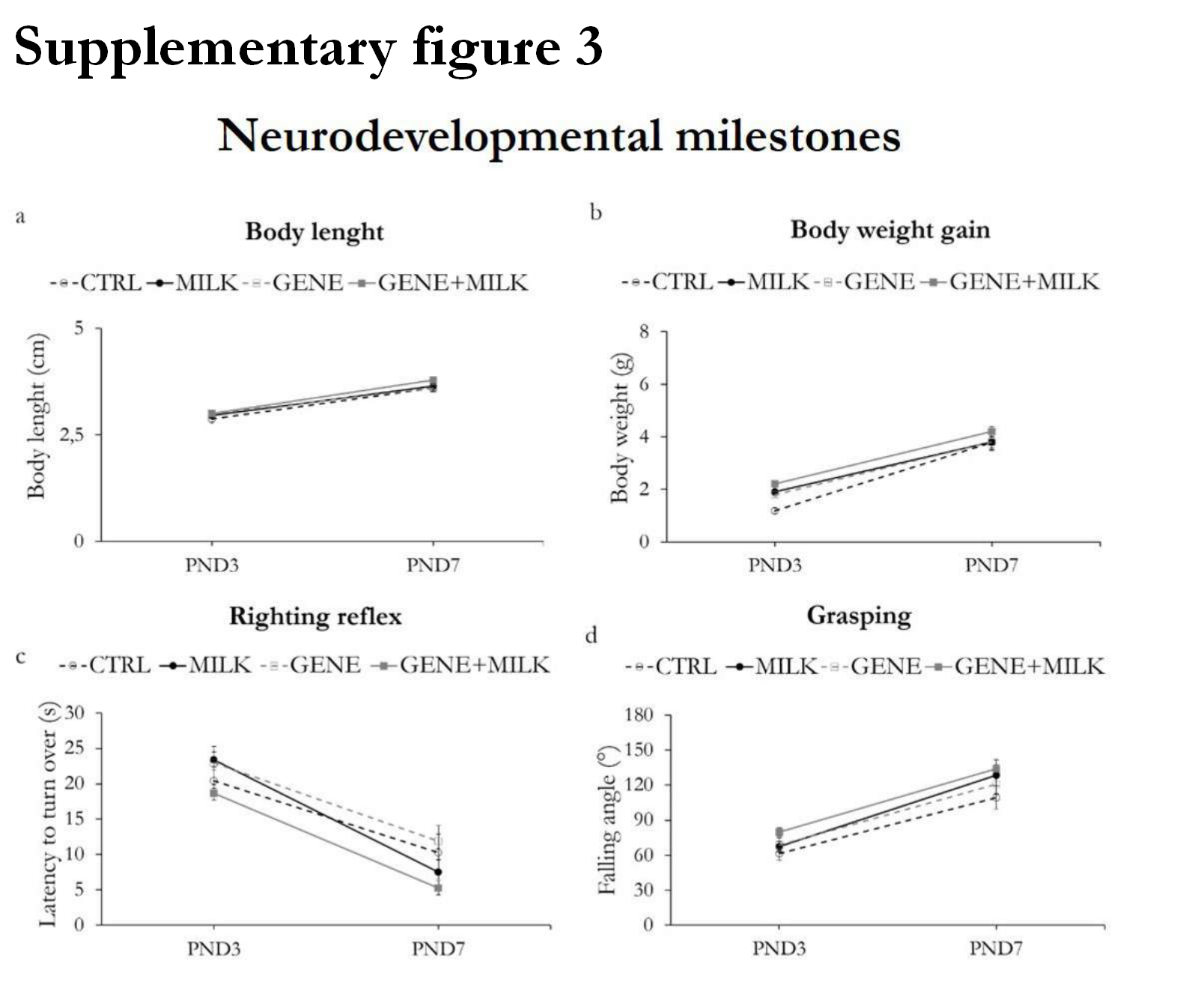

Supplement: Supplementary file 4 — Supplementary figure 3 [file 41380_2021_1054_MOESM4_ESM.tif]

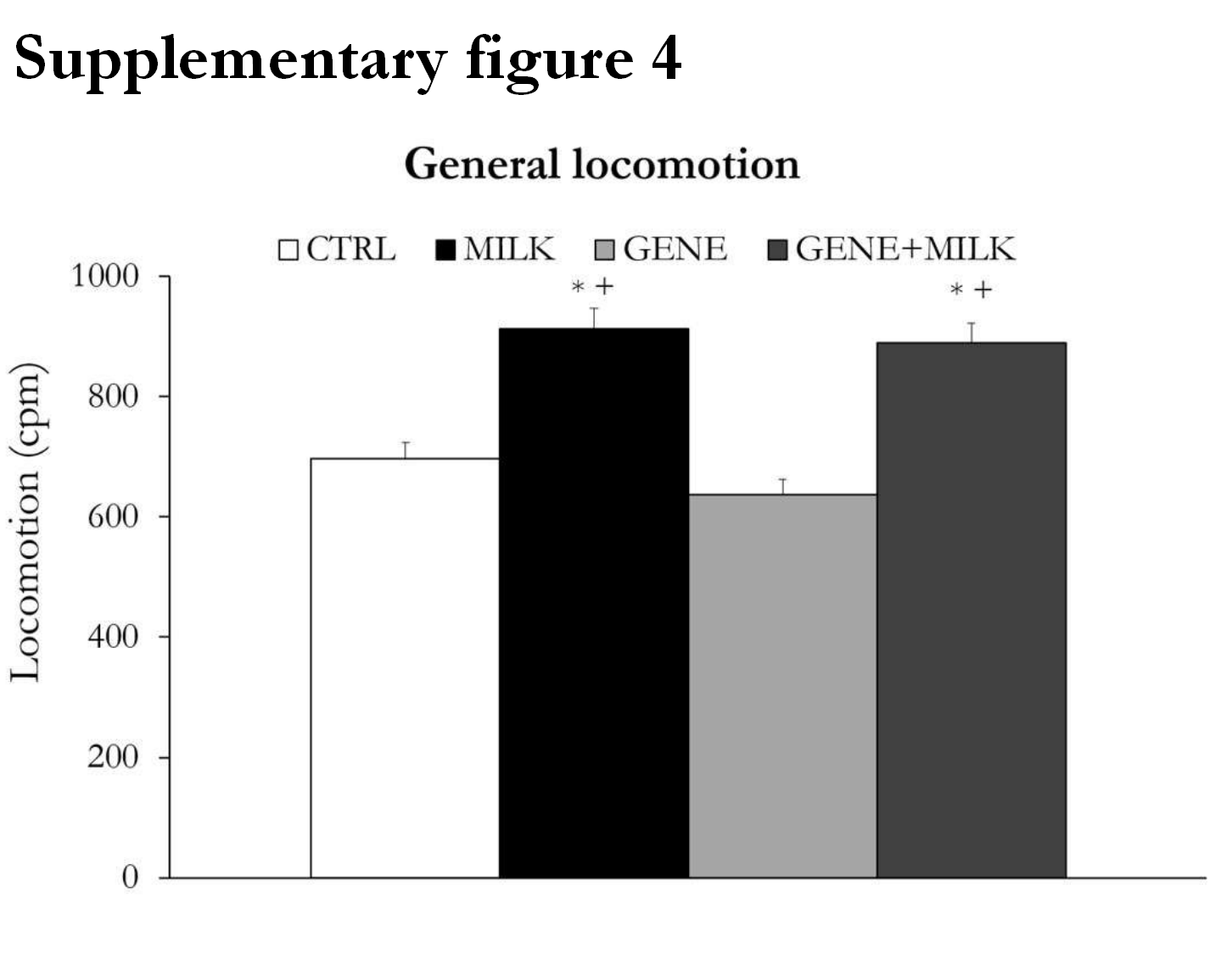

Supplement: Supplementary file 5 — Supplementary figure 4 [file 41380_2021_1054_MOESM5_ESM.tif]

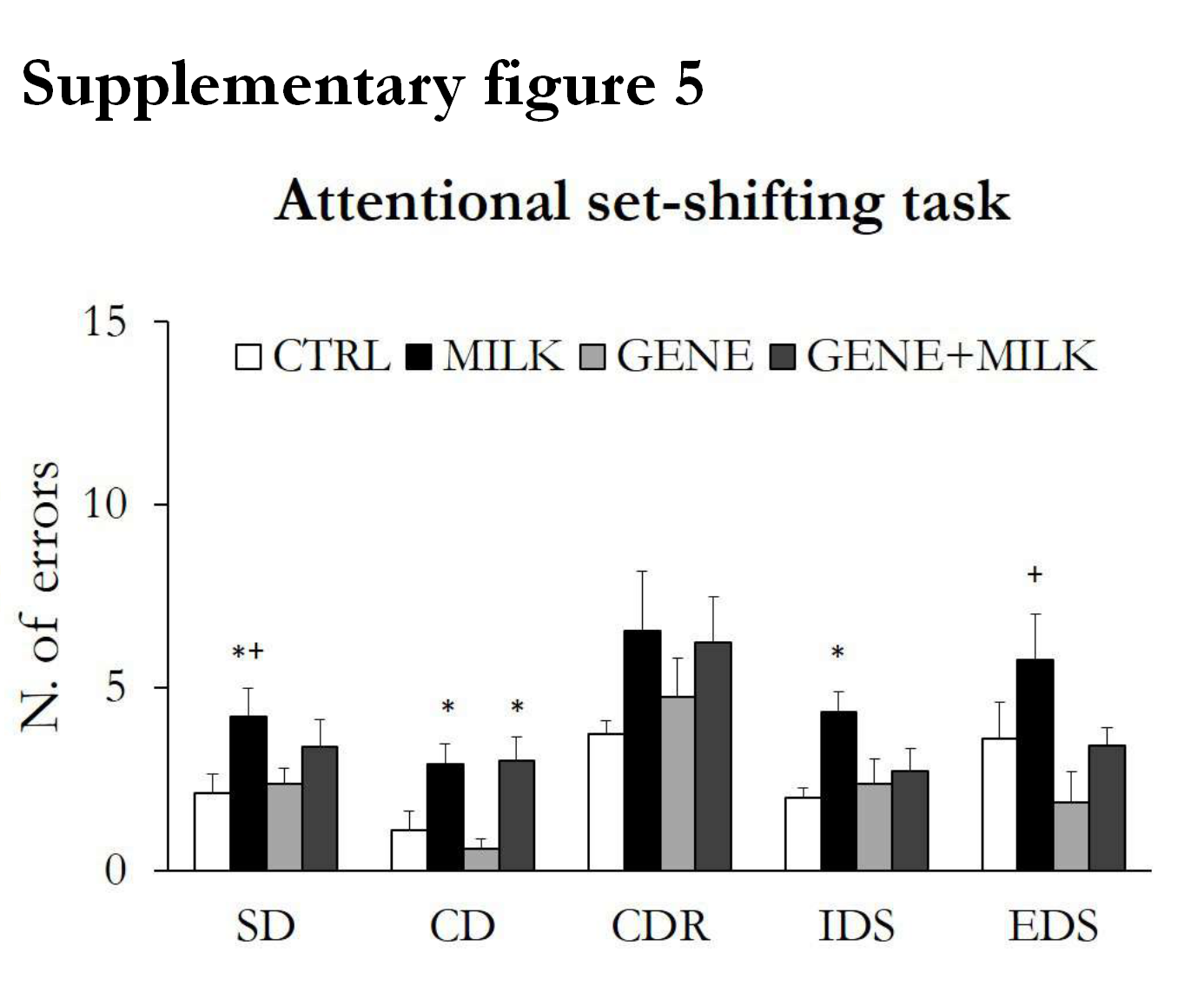

Supplement: Supplementary file 6 — Supplementary figure 5 [file 41380_2021_1054_MOESM6_ESM.tif]

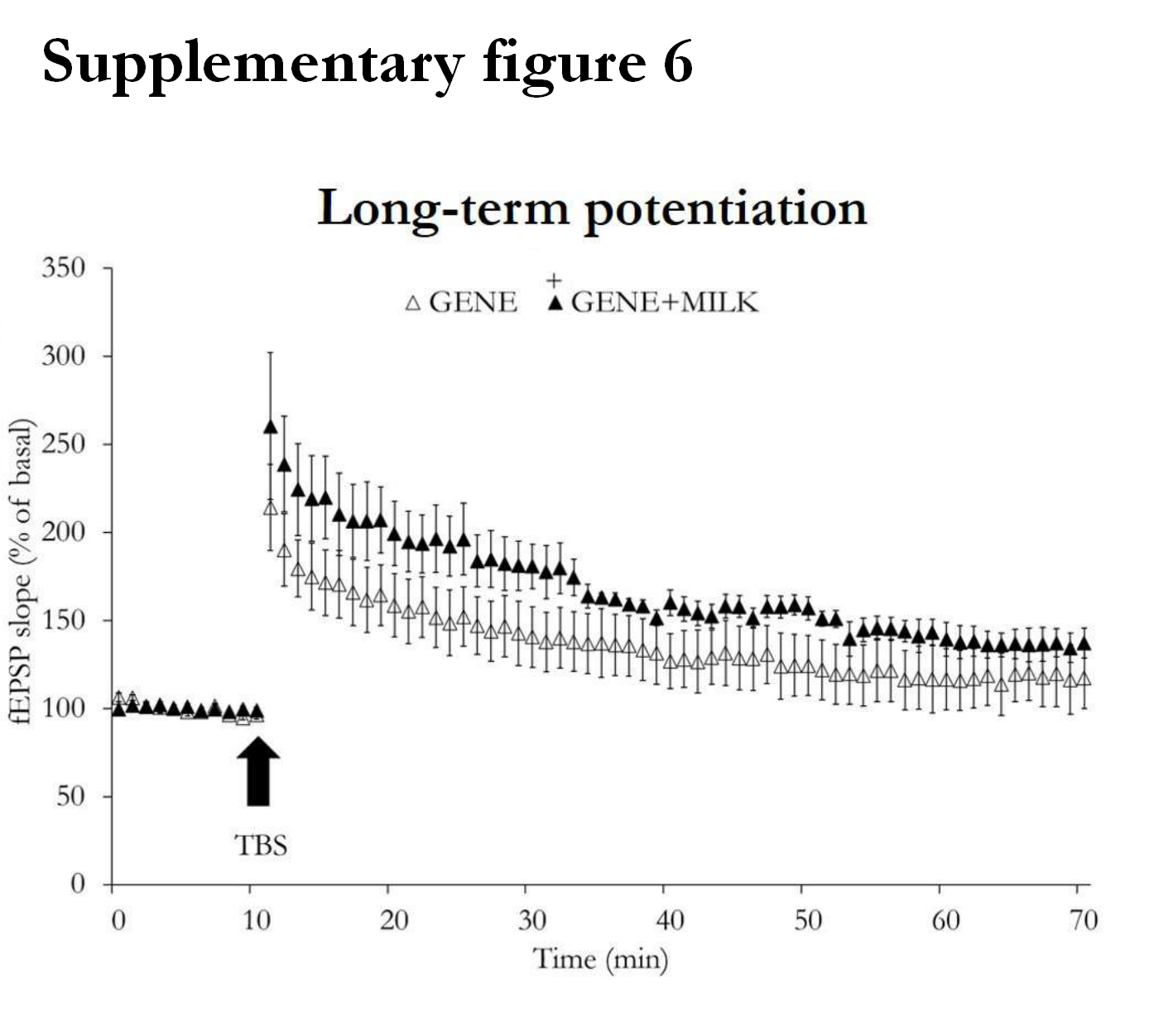

Supplement: Supplementary file 7 — Supplementary figure 6 [file 41380_2021_1054_MOESM7_ESM.tif]
